# DNAJC13 localization to endosomes is opposed by its J domain and its disordered C-terminus

**DOI:** 10.1091/mbc.E24-12-0575

**Published:** 2025-07-30

**Authors:** Hayden Adoff, Brandon Novy, Emily Holland, Braden T Lobingier

**Affiliations:** 1 Department of Chemical Physiology and Biochemistry, Oregon Health & Science University, Portland, OR 97239, USA

## Abstract

Endosomes are a central sorting hub for membrane cargos. DNAJC13/RME-8 plays a critical role in endosomal trafficking by regulating the endosomal recycling and degradative pathways. DNAJC13 localizes to endosomes through its N-terminal Pleckstrin Homology (PH)-like domain, which binds endosomal phosphatidylinositol-3-phosphate (PI(3)P). However, little is known about how DNAJC13 localization is regulated. Here, we show that two regions within DNAJC13, its J domain and disordered C-terminus, act as negative regulators of its PH-like domain. Using a structure-function approach, we map these control points to a conserved YLT motif in the disordered C-terminus as well as the catalytic HPD triad in its J domain. Mutation of either motif enhances DNAJC13 endosomal localization in cells and increases binding to PI(3)P *in vitro*, and overexpression of these mutants causes multiple defects in endosome function including endosomal clustering and loss of recycling of a membrane protein cargo. Mechanistically, the enhanced endosomal localization of DNAJC13 requires its N-terminal PH-like domain, and we show that the PH-like domain of DNAJC13 binds PI(3)P weakly in isolation and requires oligomerization for efficient PI(3)P binding and endosomal localization. Together, these results demonstrate that interaction between DNAJC13 and PI(3)P serves as a molecular control point for regulating DNAJC13 localization to endosomes.

## Introduction

Endosomes function as critical sorting hubs in the cell where membrane proteins are selectively sorted for degradation at the lysosome or for recycling to the Golgi or plasma membrane (Cullen and Steinberg, 2018). To achieve this function, endosomes host multiple proteins and protein complexes—spatially restricted into degradative and recycling subdomains—which select membrane protein cargos for trafficking to distinct destinations (Huotari and Helenius, 2011; Cullen and Steinberg, 2018). The recycling subdomain is marked by proteins which assist in removal of proteins from the maturing endosomal system, including sorting nexins like SNX1, the Retromer complex, and the actin nucleating WASH complex (Fokin and Gautreau, 2021). In contrast, the degradative subdomain is marked by proteins, including clathrin and the ESCRT complexes which concentrate ubiquitinated membrane cargos for sorting to the lysosome (Hurley and Emr, 2006; Vietri et al., 2020). Underscoring the fundamental role of this cellular task, mutations in endosomal sorting proteins have been linked to a variety of human diseases (Maxfield, 2014; Kaur and Lakkaraju, 2018).

DNAJC13 and its *Caenorhabditis elegans* ortholog RME-8 are endosomal proteins that play a critical role in this cargo sorting process (Zhang et al., 2001; Chang et al., 2004; Girard et al., 2005; Fujibayashi et al., 2008). DNAJC13 is the only known endosomal protein containing a DnaJ domain, and its interaction with the constitutively expressed heat shock protein 70 (HSC70) regulates the turnover of endosomal proteins including SNX1 and clathrin (Chang et al., 2004; Girard et al., 2005; Popoff et al., 2009; Shi et al., 2009; Freeman et al., 2014). Consequently, loss of DNAJC13 results in missorting of both degrading and recycling cargos like the cation independent mannose-6-phosphate receptor, MIG-14/Wntless, Notch, the delta opioid receptor, and the beta-2 adrenergic receptor (β2AR) (Popoff et al., 2009; Shi et al., 2009; Gomez-Lamarca et al., 2015a; Novy et al., 2024). DNAJC13 is also implicated in endosomal homeostasis, as loss of DNAJC13 causes aberrant enlargement of endosomes in human and *Drosophila melanogaster* cells, and loss of *C. elegans* RME-8 causes intermixing of normally spatially restricted endosomal subdomains (Gomez-Lamarca et al., 2015a; Norris et al., 2017; Novy et al., 2024). Consistent with a critical role in endosomal function, homozygous knockout of DNAJC13 in mice is embryonic lethal and heterozygous mice have increased circulating bilirubin levels and reduced mean corpuscular hemoglobin (Groza et al., 2023). Additionally, point mutations in DNAJC13 have potential links to neurological diseases in humans including essential tremor and Parkinson’s disease (Vilariño-Güell et al., 2014; Rajput et al., 2015; Deng et al., 2016; Deng and Siddique, 2017; Farrer et al., 2017).

Like other endosomal proteins, DNAJC13 must first localize to endosomes to function. Localization of DNAJC13 to endosomes is driven by its N-terminal Pleckstrin Homology (PH)-like domain which can directly bind to the endosomal enriched phosphoinositide, phosphatidylinositol-3-phosphate (PI(3)P) (Xhabija and Vacratsis, 2015). Deletion of the DNAJC13 N-terminus shifts its localization from endosomes to the cytoplasm, and point mutations within its N-terminal PH-like domain inhibit its endosomal localization in cells and block PI(3)P binding *in vitro* (Fujibayashi et al., 2008; Freeman et al., 2014; Xhabija and Vacratsis, 2015). However, what regulates DNAJC13 localization to endosomes, and PI(3)P binding, is unknown. While DNAJC13 has been shown to bind other endosomal proteins including SNX1 and FAM21, these do not control its localization (Freeman et al., 2014; Xhabija and Vacratsis, 2015). One common mechanism that regulates endosomal proteins that bind to PI(3)P is that many have low affinity for PI(3)P as isolated monomers and have improved affinity for PI(3)P *in vitro,* and increased localization to endosomes in cells, when oligomerized (Klein et al., 1998; Hayakawa et al., 2004). This multivalency requirement for PI(3)P binding has been most clearly demonstrated for EEA1, where structural studies have shown that EEA1 homodimerization allows the FYVE domain from each monomer to simultaneously engage PI(3)P (Dumas et al., 2001). However, it is unknown if DNAJC13 has a multivalency requirement for PI(3)P-binding and if regions outside its N-terminus affect its ability to localize to endosomes.

Recent advances in structural modeling using AlphaFold (AF), and newer versions AF2 and AF3, have opened the door to creating specific, testable hypotheses about the structure-function relationship of a protein. We noted that the AF model of DNAJC13 predicted the last 45 amino acids of its C-terminus to be an intrinsically disordered region (IDR) (Jumper et al., 2021; Varadi et al., 2022). As IDRs have a known role in protein regulation and autoinhibition, we hypothesized that this region may play a role in regulation of DNAJC13 function (Fenton et al., 2023). Thus, we set out to determine how localization of DNAJC13 to endosomes is regulated and how its distinct domains—including its N-terminal PH-like domain, J domain, and its disordered C-terminus—affect its localization.

## Results

### DNAJC13 disordered C-terminus controls its localization

**Figure 1.**
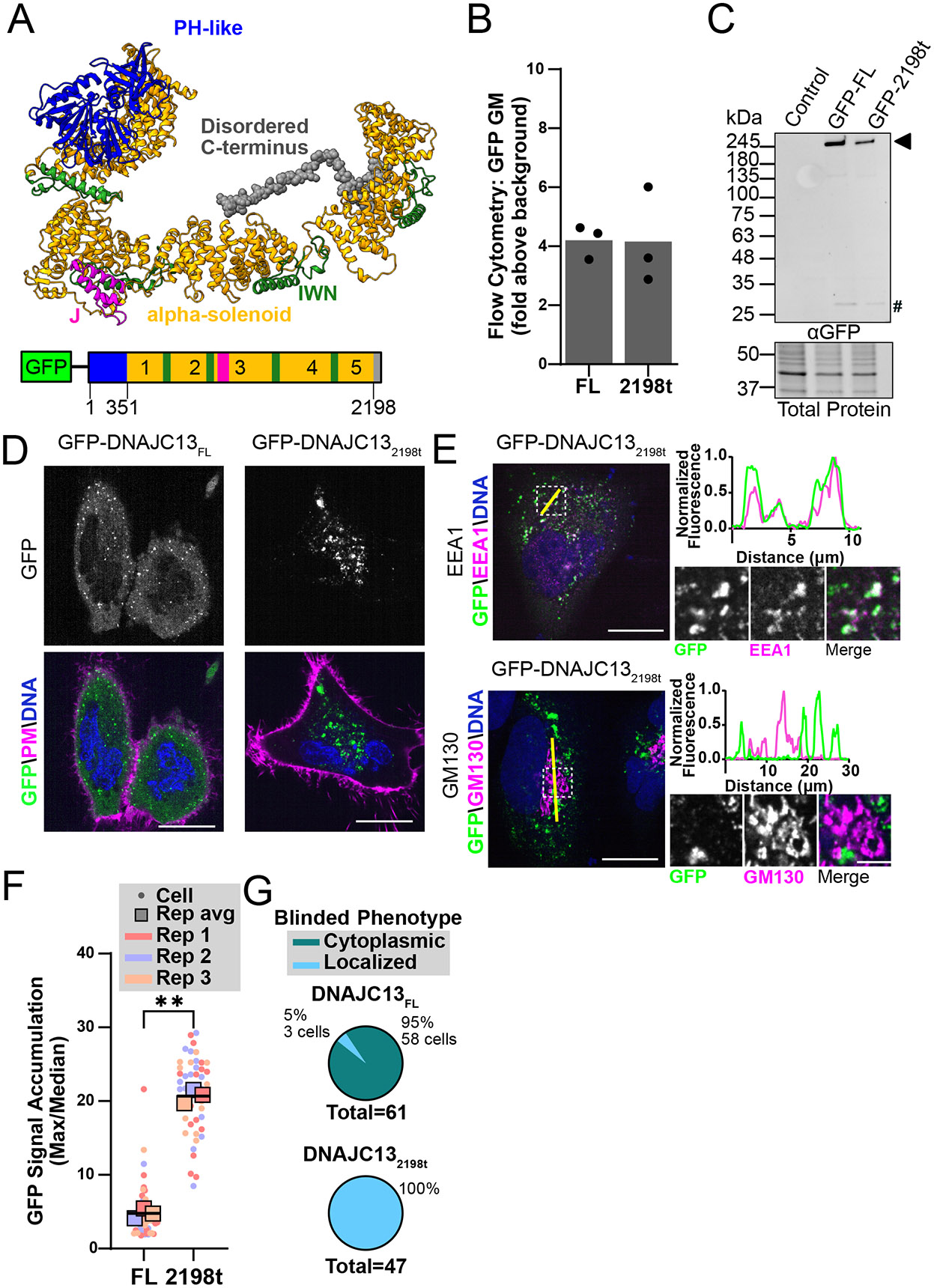
DNAJC13 disordered C-terminus controls its localization. ***A***, AlphaFold2.0 structure for human DNAJC13 (AF-O75165-F1-v4) (top) colored by domain (bottom), including the N-terminal PH-like domain (blue), five alpha solenoids (yellow) separated by repeating IWN motifs with potential regulatory function (Zhang et al., 2001; Norris et al., 2022; Varadi et al., 2022) (dark green), a J domain (magenta) and C-terminus (grey, space filled residues). ***B***, Flow cytometry-based expression analysis of GFP-DNAJC13 constructs transfected into HeLa cells, assessed by geometric mean of GFP channel and displayed as fold above background signal from untransfected cells (n=3 biological replicates, bar represents mean). ***C***, Representative western blot of extracts from transiently expressed GFP-DNAJC13 constructs in HeLa cells, with a nontransfected control (Control), anti-GFP immunoblot (top) and total protein loading control (bottom), (n=3 biological replicates). The arrowhead marks GFP-DNAJC13 and the # marks free GFP. ***D***, Live spinning disk confocal microscopy images of GFP-DNAJC13 constructs in HeLa cells. Imaged with CellMask plasma membrane stain (magenta) and Hoechst DNA stain (blue) (scale bars = 20 μm) (representative example from n=3 biological replicates). ***E***, Fixed immunofluorescence microscopy image of GFP-DNAJC13_2198t_ expressed in HeLa cells. Imaged with anti-GFP (Green), DAPI DNA stain (blue), and endosomal marker anti-EEA1 (magenta, top) or Golgi marker GM130 (magenta, bottom). Insets shown to the right (scale bar = 20 μm, 5 μm in inset), (representative example from n=3 biological replicates). Line-scans (yellow line) showing normalized fluorescence intensity of GFP (green) and EEA1 (magenta) or GM130 (magenta) signal are plotted along the line (right). ***F***, SuperPlot of cellular GFP signal accumulation metric (maximal GFP signal divided by median GFP signal) of individual cells with single cell data shown in circles and biological replicate averages plotted in squares, colored by replicate (Lord et al., 2020). The black bars indicate the means of three biological replicate averages, with statistics performed on these averages (n=3 biological replicates, paired two-tailed t-test comparing biological replicate averages, p=0.0022). ***G***, Blinded analysis of live cell microscopy images of cells expressing DNAJC13_FL_ and DNAJC13_2198t_ for phenotype either being predominantly cytoplasmic (green) or predominantly localized to vesicles with little to no cytoplasmic signal (blue). The same cells analyzed by GFP signal accumulation metric in F were independently scored for localization.

We noted that the AF2 model of human DNAJC13 predicted the final 45 amino acids of its C-terminus to be an IDR (Figure 1A). We next examined two other structural prediction programs, the disorder predictor JRonn and five additional AF3 models, which also predicted the C-terminus of DNAJC13 to be disordered (Figure S1A) (Waterhouse et al., 2009; Troshin et al., 2011; Abramson et al., 2024). As IDRs commonly serve regulatory functions, we hypothesized that the disordered C-terminus of DNAJC13 could affect its localization to endosomes (Fenton et al., 2023).

To test this hypothesis, we designed several DNAJC13 constructs using a similar N-terminal GFP tagging scheme to that used by others previously (Fujibayashi et al., 2008; Xhabija et al., 2011; Freeman et al., 2014; Yoshida et al., 2018): full-length GFP-DNAJC13 (DNAJC13_FL_) or GFP-DNAJC13 lacking its last 45 amino acids (DNAJC13_2198t_). We first analyzed the relative expression of these constructs by flow cytometry and found they express at similar levels (Figure 1B). Additionally, by western blot we saw minimal evidence of proteolysis and liberation of free GFP (Figures 1C, S1B). We also examined full-length DNAJC13 with a C-terminal GFP (DNAJC13_FL_-GFP) but found that it expressed poorly (less than 10% of the expression of DNAJC13 with an N-terminal GFP tag), which did not allow for further analysis (Figure S1C).

We then sought to determine the localization of these GFP-DNAJC13 constructs in cells using live microscopy and found, similar to previous observations, that overexpressed DNAJC13_FL_ localized to both the cytoplasm and vesicles (Figures 1D) (Fujibayashi et al., 2008; Freeman et al., 2014). Strikingly, DNAJC13_2198t_ was highly localized to vesicles with minimal cytoplasmic background (Figure 1D). As DNAJC13/RME-8 localizes to early endosomes, we turned to immunofluorescence microscopy to determine the identity of the DNAJC13-positive structures (Zhang et al., 2001; Girard et al., 2005; Fujibayashi et al., 2008; Shi et al., 2009; Xhabija and Vacratsis, 2015; Novy et al., 2024). Using the early endosomal marker EEA1 and the Golgi marker GM130, we confirmed that GFP-DNAJC13-positive vesicles are indeed early endosomes (Figures 1E, Figure S1D).

To further characterize the enhanced endosomal localization of DNAJC13_2198t_, we performed two orthogonal methods of analysis. First, to quantitatively differentiate between cytoplasmic and localized distribution of DNAJC13, we devised a GFP signal accumulation metric. In this metric, the maximal fluorescence within a cell is divided by the median fluorescence across the entire cell; thus, a score of 1 would indicate the signal is homogeneous throughout the cell, much like free GFP, while a higher score indicates a localized protein with non-homogenous distribution. Using the quantitative GFP accumulation metric, we found that DNAJC13_2198t_ had ~4.3-fold higher score than DNAJC13_FL_ (Figure 1F). Second, we performed blinded qualitative analysis to assess GFP signal in cells as either “cytoplasmic,” and thus containing highly cytoplasmic GFP in addition to GFP-positive vesicles, or “localized,” and thus containing GFP predominantly localized to vesicles with little to no cytoplasmic GFP. Blinded qualitative analysis found that only 5% of cells expressing DNAJC13_FL_ had a predominantly vesicular localization, whereas all DNAJC13_2198t_ expressing cells examined showed a localized phenotype (Figure 1G). Lastly, since these independent methods of analysis were performed on the same populations of cells, we could compare the metrics by overlaying the blinded analysis onto GFP signal accumulation score for each cell. This overlay analysis showed a strong agreement between the metrics, and the three DNAJC13_FL_-expressing cells which scored as localized in blinded phenotypic analysis also had the highest scores in the signal accumulation metric (Figure S1E). Thus, by both live and fixed imaging, we found that removal of the disordered C-terminus of DNAJC13 enhanced its localization to endosomes.

### YLT residues in DNAJC13 C-terminus control endosomal localization

**Figure 2.**
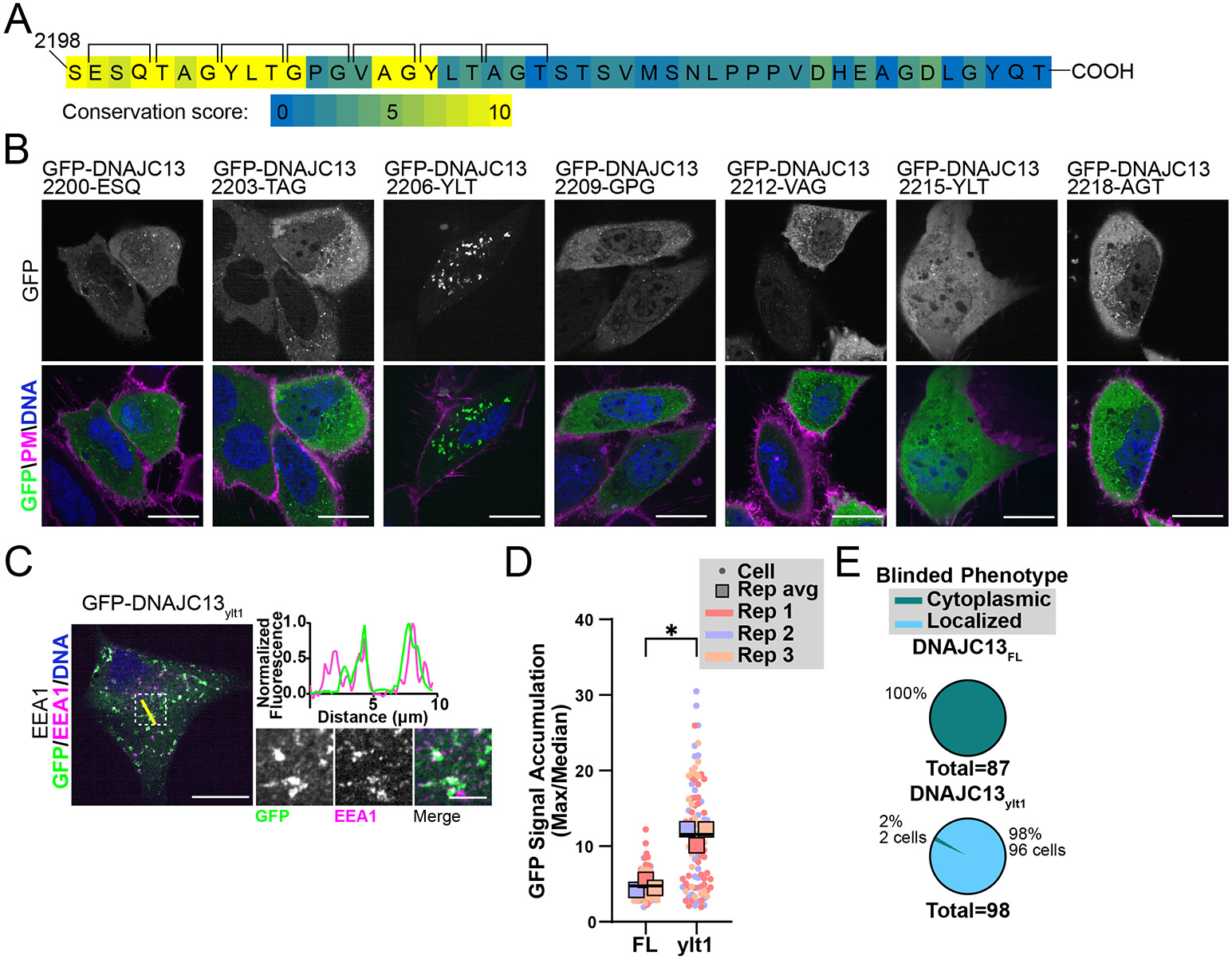
YLT residues in C-terminus control endosomal localization. ***A***, Relative conservation analysis of the DNAJC13 C-terminal IDR (45 residues) amongst all orthologues in Ensemble vertebrate (plus *C. elegans and D. melanogaster*) database (less conserved = more blue; more conserved = more yellow). Brackets above indicate regions for triplet alanine scanning. ***B***, Live spinning disk confocal microscopy images of triplet scan mutants, expressed in HeLa cells. Imaged with CellMask plasma membrane stain (magenta) and Hoechst DNA stain (blue) (scale bar = 20 μm) (representative example from n=3 biological replicates). ***C***, Fixed immunofluorescence microscopy image of GFP-DNAJC13_ylt1_ expressed in HeLa cells. Imaged with anti-GFP (green), endosomal marker anti-EEA1 (magenta), and DAPI DNA stain (blue) with insets shown to the right (scale bar = 20 μm, 5 μm in inset), (representative example from n=3 biological replicates). A line-scan (yellow line) showing normalized fluorescence intensity of GFP (green) and EEA1 (magenta) signal are plotted along the line (right). ***D***, SuperPlot of cellular GFP signal accumulation metric of individual cells with single cell data shown in circles and biological replicate averages plotted in squares, colored by replicate. The black bars indicate the means of three biological replicate averages, with statistics performed on these averages (n=3 biological replicates, paired two-tailed t-test comparing biological replicate averages, p=0.0258). ***E***, Blinded analysis of live cell microscopy images of cells expressing DNAJC13_FL_ or DNAJC13_ylt1_ for phenotype either being predominantly cytoplasmic (green) or predominantly localized to vesicles with little to no cytoplasmic signal (blue). The same cells analyzed by GFP signal accumulation metric in D were independently scored for localization, and represents a different population of cells expressing DNAJC13_FL_ from those shown in Figure 1G.

We next asked which part of the DNAJC13 C-terminus was necessary to control its localization to endosomes. We examined the evolutionary conservation of the last 45 amino acids of DNAJC13—those predicted to be disordered—by calculating a relative conservation score using the Ensembl database of vertebrate orthologues (plus *C. elegans and D. melanogaster*) (Waterhouse et al., 2009; Harrison et al., 2024). We found the first 17 amino acids to be more highly conserved than the final 28 (Figure 2A). Consequently, we focused on this conserved region and used alanine scanning to mutate blocks of three residues at a time to probe for which amino acids were important in controlling DNAJC13 localization (Figure 2A, brackets). Analysis of these constructs showed they were expressed at similar levels without significant proteolysis (Figures S2A-B).

Using live cell microscopy, we assessed these constructs for localization and found that only one triple alanine mutant, DNAJC13_ylt1_ (2206-YLT-AAA) increased vesicular accumulation of DNAJC13 (Figure 2B). We again confirmed endosomal localization of DNAJC13_ylt1_ with immunofluorescence imaging using EEA1 and GM130 probes (Figures 2C, S2C). Interestingly, we noticed a second occurrence of a YLT sequence in the DNAJC13 C-terminus (DNAJC13_ylt2_, 2215-YLT), but observed no overt phenotype upon mutation (Figure 2B). We then utilized the GFP signal accumulation metric to quantify the signal distribution of DNAJC13_ylt1_ in cells and found a ~2.4-fold increase over DNAJC13_FL_ (Figure 2D). Consistent with this observation, new blinded analysis comparing to DNAJC13_FL_ to DNAJC13_ylt1_ again showed that DNAJC13_FL_ is predominantly cytoplasmic while most DNAJC13_ylt1_-expressing cells scored as predominantly localized to endosomes with little or no cytoplasmic background (98% of cells) (Figure 2E). We noted a lower population of DNAJC13_ylt1_-expressing cells in GFP signal accumulation metric (~30% of cells) with scores less than seven. We considered that these cells might represent a different DNAJC13 localization phenotype, but direct comparison of our different analysis methods showed that most of the DNAJC13_ylt1_ expressing cells in this lower population scored as localized (Figure S2D). In the process of analyzing this lower population, we noted these cells tended to express DNAJC13 at lower levels but occur in the same field of view as cells expressing DNAJC13 at higher levels, and this observation was supported by signal-to-noise analysis of the entire population (Figure S2E-F). We think this is highly consistent with our transient transfection workflow which results in differential expression of DNAJC13 between individual cells in the population. Thus, while we cannot rule out that cells expressing DNAJC13_ylt1_ at very low levels might show a distinct pattern of localization, our data support the conclusion that most cells—high and low expressing—show a localized phenotype. Together, our data suggests a model in which the disordered C-terminus, driven primarily by a YLT sequence (Human: 2206-2208) regulates DNAJC13’s endosomal localization.

### J domain co-regulates DNAJC13 localization

We next asked how protein-protein interactions could contribute to control of DNAJC13 localization to endosomes. The three major known interacting proteins of DNAJC13/RME-8, which have been identified by co-immunoprecipitation or yeast two-hybrid, are FAM21, SNX1, and HSC70 (Chang et al., 2004; Girard et al., 2005; Shi et al., 2009; Freeman et al., 2014). While FAM21 and SNX1 do not affect DNAJC13 localization to endosomes, it has not been investigated if HSC70 plays any role in regulation of the subcellular localization of DNAJC13 (Freeman et al., 2014; Xhabija and Vacratsis, 2015). To test the hypothesis that HSC70 could affect DNAJC13 localization in cells, we created constructs in which the HPD residues in the J domain, which are critical for binding HSC70 and stimulating HSC70 ATPase activity, were mutated to alanines (termed DNAJC13_hpd_ and dual mutants, DNAJC13_2198t(hpd)_ or DNAJC13_ylt1(hpd)_) (Chamberlain and Burgoyne, 1997; Morgan et al., 2001; Yan et al., 2002; Tummala et al., 2016). These constructs expressed at similar levels with minimal proteolysis (Figures S3A-D).

**Figure 3.**
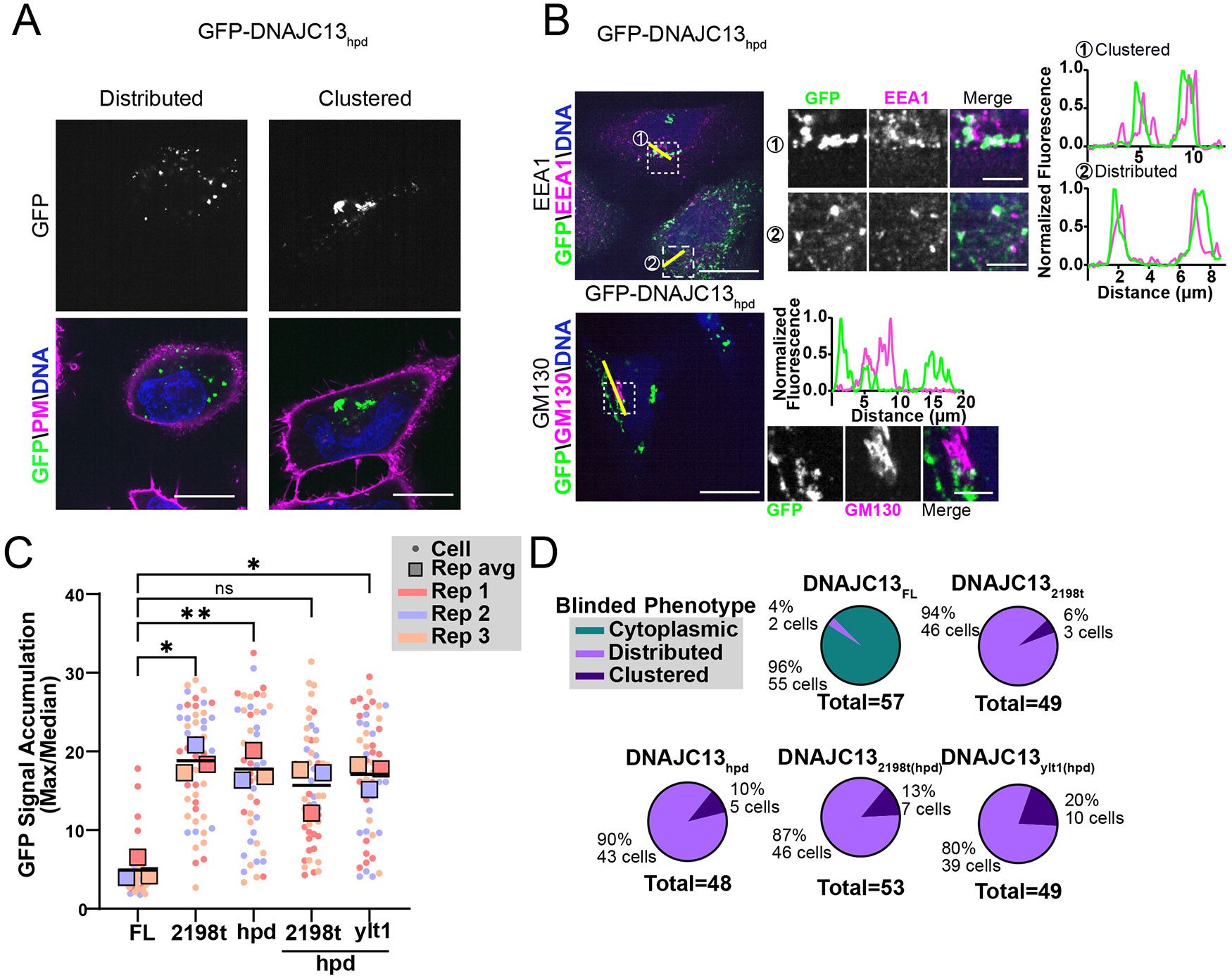
J domain co-regulates DNAJC13 localization. ***A***, Live cell spinning disk confocal microscopy images of GFP-DNAJC13_hpd_ in HeLa cells exhibiting distributed (left) and clustered (right) endosomes. Imaged with CellMask plasma membrane stain (magenta) and Hoechst DNA stain (blue) (scale bar = 20 μm) (phenotypic representative examples from n=3 biological replicates). ***B***, Fixed immunofluorescence microscopy image of GFP-DNAJC13_hpd_ expressed in HeLa cells. Imaged with anti-GFP (green), DAPI DNA stain (blue), and endosomal marker anti-EEA1 (magenta, left) or Golgi marker anti-GM130 (magenta, right). Insets shown to the right (scale bar = 20 μm, 5 μm in insets), (representative example from n=3 biological replicates). Line-scans (yellow lines) for each inset showing normalized fluorescence intensity of GFP (green) and EEA1 (magenta) or GM130 (magenta) signal are plotted along the lines (right). ***C***, SuperPlot of cellular GFP signal accumulation metric of individual cells with single cell data shown in circles and biological replicate averages plotted in squares, colored by replicate. The black bars indicate the means of three biological replicate averages, with statistics performed on these averages (n=3 biological replicates, one-way paired ANOVA comparing biological replicate averages with Dunnett’s multiple comparisons corrections, all against DNAJC13_FL_, p=0.0255 (DNAJC13_2198t_), 0.0018 (DNAJC13_hpd_), 0.1154 (DNAJC13_2198t(hpd)_), 0.0135 (DNAJC13_ylt1(hpd)_). ***D***, Blinded analysis of live cell microscopy images of cells expressing DNAJC13_FL_, DNAJC13_2198t_, DNAJC13_hpd_, DNAJC13_2198t(hpd)_, and DNAJC13_ylt1(hpd)_ for phenotype being either: largely cytoplasmic (green), localized to distributed endosomes (light purple), or localized to endosomes clustered to a perinuclear region (dark purple). The same cells analyzed by GFP signal accumulation metric in C were independently scored for localization and represent a different population of cells expressing DNAJC13_FL_ and DNAJC13_2198t_ from Figure 1G and Figure 2E.

Similar to DNAJC13_2198t_, DNAJC13_hpd_ showed strong localization to endosomes with little DNAJC13 residing in the cytoplasm (Figures 3A,B). Interestingly, we observed that in a subset of the DNAJC13_hpd_ expressing cells, the GFP-DNAJC13-positive endosomes clustered in a perinuclear region that was distinct from the Golgi (Figures 3A,B). We observed a similar phenomenon in the double mutants, DNAJC13_2198t(hpd)_ and DNAJC13_ylt1(hpd)_ (Figures S4A-C). Notably, endosomal clustering has been observed upon manipulation of proteins which either physically- (WASH complex) or functionally- (clathrin) interact with DNAJC13 (Bennett et al., 2001; Gomez et al., 2012).

To further examine how mutation of the DNAJC13 disordered C-terminus and J domain, either individually or in combination, affect its localization, first we analyzed cells expressing the DNAJC13 mutants with our GFP signal accumulation metric. We found that all four DNAJC13 mutants showed a strong increase in signal accumulation metric, consistent with an increase in endosomal localization (Figure 3C; fold above DNAJC13_FL_: DNAJC13_2198t_ =3.8, DNAJC13_hpd_ =3.6, DNAJC13_2198t(hpd)_=3.2, DNAJC13_ylt1(hpd)_=3.5). While the double mutant DNAJC13_2198t(hpd)_ did not reach statistical significance, this is likely due to a single replicate which had on average a higher portion of cells in the lower expressing population, which could be identified in a signal:noise analysis (Figure S4D).

To assess distribution of DNAJC13 positive endosomes between the distributed and clustered phenotypes, we performed new blinded analysis in which cells were scored for GFP signal as being predominantly cytoplasmic, localized to distributed vesicles, or localized to clustered vesicles, defined as signal coming from three or fewer contiguous structures. We found no instances of the endosomal clustering phenotype in cells expressing GFP-DNAJC13_FL_, while cells expressing DNAJC13_2198t_ or DNAJC13_hpd_ showed similar proportions of distributed and clustered endosomal vesicles (Figure 3D, DNAJC13_2198t_: 6% clustered; DNAJC13_hpd_: 10% clustered). Interestingly, the double mutants showed a larger percentage of cells with the endosomal clustering phenotype, suggesting the potential for an additive effect of simultaneous mutation to the DNAJC13 J domain and disordered C-terminus (Figure 3D, DNAJC13_2198t(hpd)_: 13% clustered; DNAJC13_ylt1(hpd)_: 20% clustered). In a cross-comparison of our blinded analysis and GFP accumulation metric, we found no correlation between clustered phenotype and signal accumulation metric (Figure S4E). This is consistent with the metric being well suited to differentiation between highly homogenous, cytoplasmic signal and highly localized signal, but not being able to distinguish types of localization. Thus, these two methods for analyzing DNAJC13 localization in cells are aligned and complementary in their overall conclusions about which DNAJC13 mutations result in increased localization to endosomes, but the blinded analysis proved more effective at identifying a sub-population of DNAJC13-expressing cells which showed an endosomal clustering phenotype.

Together, these observations suggest that there are two control points for DNAJC13 localization to endosomes: a YLT motif in the C-terminus and its J domain. Additionally, our observations suggest that overexpression of DNAJC13 carrying these activating mutations can act in a dominant negative manner to affect endosomal distribution in the cell, which is similar to previous observations following disruption of WASH or clathrin function (Bennett et al., 2001; Gomez et al., 2012).

### Disordered C-terminus and J domain act through PH-like domain to enhance PI(3)P binding

We next sought to analyze the mechanism by which the J domain and C-terminal mutants enhance DNAJC13 localization to endosomes. DNAJC13 is known to localize to endosomes through a PH-like domain in its N-terminus (first ~100 residues) (Xhabija et al., 2011; Xhabija and Vacratsis, 2015). Thus, we considered the possibility that the J domain and C-terminal IDR were modulating the ability of the N-terminal PH-like domain to bind to PI(3)P.

**Figure 4.**
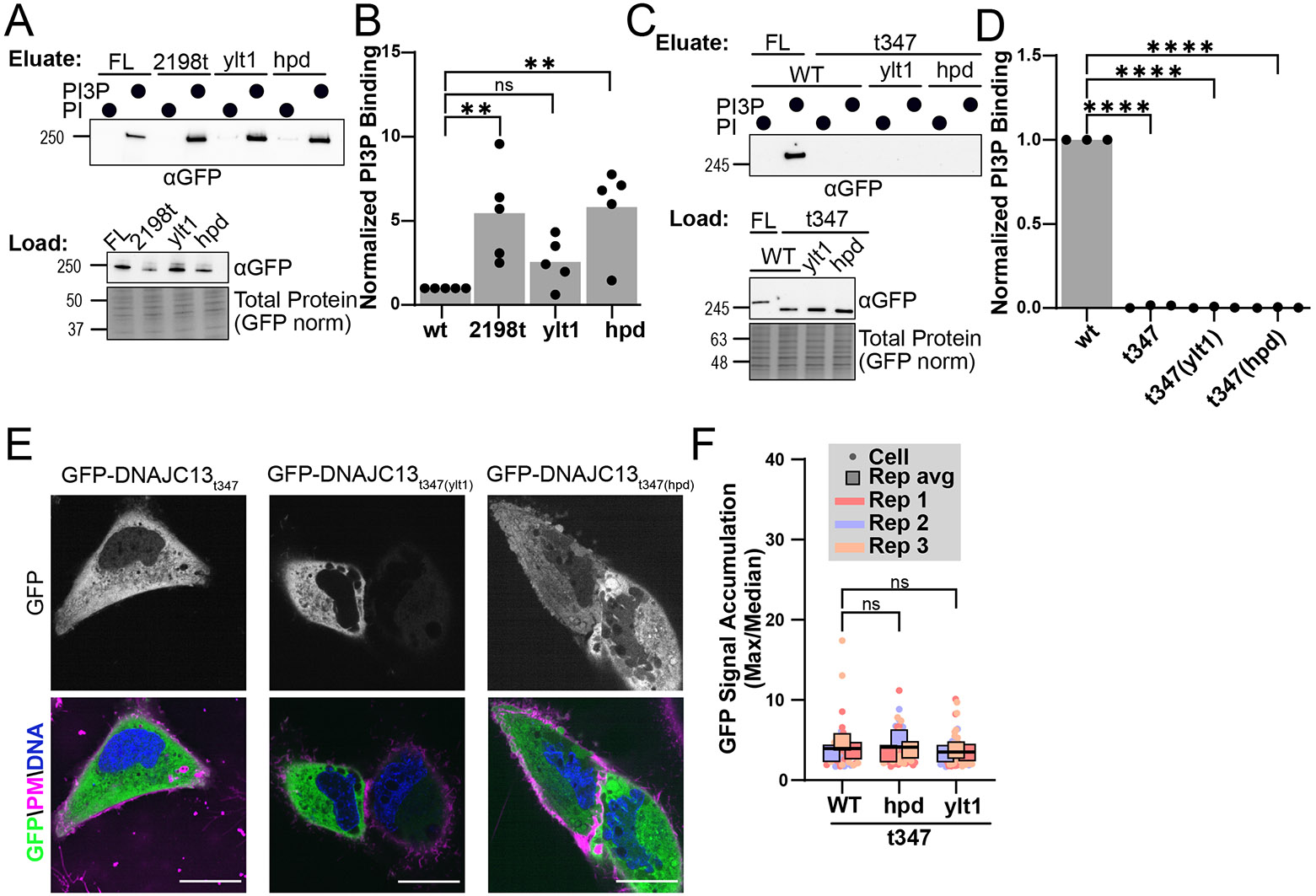
C-terminus and J domain act through PH-like domain to enhance PI(3)P binding. ***A***, Western blots of PIP resin eluates for DNAJC13_FL_ and activating mutants. GFP-DNAJC13_FL_, GFP-DNAJC13_2198t_, GFP-DNAJC13_ylt1_, and GFP-DNAJC13_hpd_ were expressed in HEK293 cells and lysates, normalized by flow cytometry for GFP expression, and were bound to PI (control) and PI(3)P decorated agarose resins. Loads and eluates were run on SDS-PAGE (load total protein stain, bottom) and immunoblotted for anti-GFP (load, middle; eluate, top). ***B***, Quantification of PI(3)P pulldowns in A, normalized to load and the full-length pulldown (n=5 biological replicates, bar represents mean, one-way unpaired ANOVA with Dunnett’s multiple comparisons corrections, all vs DNAJC13_FL_, p= 0.0085 (DNAJC13_2198t_), 0.4915 (DNAJC13_ylt1_), 0.0046 (DNAJC13_hpd_)). ***C***, Western blots of PIP resin eluates of DNAJC13 lacking PH-like domains. GFP-DNAJC13_FL_, GFP-DNAJC13_t347_, GFP-DNAJC13_t347(ylt1)_, and GFP-DNAJC13_t347(hpd)_ were expressed in HEK293 cells and lysates, normalized by flow cytometry for GFP expression, were bound to PI (control) and PI(3)P decorated agarose resins. Loads and eluates were run on SDS-PAGE (load total protein stain, bottom) and immunoblotted for anti-GFP (load, middle; eluate, top). ***D***, Quantification of PI(3)P pulldowns in C, normalized to load and the full-length pulldown (n=3 biological replicates, bar represents mean, one-way paired ANOVA with Dunnett’s multiple comparisons corrections, all against DNAJC13_FL_, p<0.0001 for all comparisons). ***E***, Live cell spinning disk confocal microscopy images of GFP-DNAJC13_t347_, GFP-DNAJC13_t34(ylt1)_, GFP-DNAJC13_t347(hpd)_ in HeLa cells. Imaged with CellMask plasma membrane stain (magenta) and Hoechst DNA stain (blue) (scale bar = 20 μm), (representative example from n=3 biological replicates). ***F***, SuperPlot of cellular GFP signal accumulation metric of individual cells with single cell data shown in circles and biological replicate averages plotted in squares, colored by replicate. The black bars indicate the means of three biological replicate averages, with statistics performed on these averages (n=3 biological replicates, one-way paired ANOVA comparing biological replicate averages with Dunnett’s multiple comparisons corrections vs DNAJC13_t347_, ns for all).

To test this, we examined binding of DNAJC13 in detergent lysates to agarose beads conjugated to PI(3)P. As had been observed previously, we found that DNAJC13_FL_ bound efficiently to PI(3)P and did not bind to the negative control, phosphatidylinositol (PI) (Figures 4A, S5A) (Xhabija et al., 2011; Xhabija and Vacratsis, 2015). We then examined the DNAJC13 mutations that enhanced its endosomal localization (DNAJC13_2198t_, DNAJC13_ylt1_, and DNAJC13_hpd_, and DNAJC13_ylt1(hpd)_) and found increased PI(3)P binding compared to DNAJC13_FL_ (Figures 4A, S5A-D). Quantification of these results showed that DNAJC13_2198t_ and DNAJC13_hpd_ bound PI(3)P decorated resins ~5-fold better than DNAJC13_FL_ (Figure 4B). Additionally, we observed a ~2-3-fold better PI(3)P binding of DNAJC13_ylt1_ or DNAJC13_ylt1(hpd)_ compared to DNAJC13_FL_, although the former did not reach statistical significance (Figure S5D, 4B). Together, these results demonstrate that mutations in DNAJC13 which increase its endosomal localization in cells also increase DNAJC13 binding to PI(3)P *in vitro*.

We next tested if the enhanced binding of DNAJC13 to PI(3)P we observed upon mutation of the J domain or C-terminus required its N-terminal PH-like domain. While previous work has shown that a single point mutations in the DNAJC13 PH-like domain blocks its binding to PI(3)P, recent AF2 analysis of DNAJC13 and RME-8 identified that its globular N-terminus (first ~340 residues) contains not one but three PH-like folds, although it is unclear if the latter two have any PI(3)P binding activity (Norris et al., 2022). Thus, to remove any potential contribution from the second and third PH-like folds to the ability of DNAJC13 to bind PI(3)P, we created DNAJC13 constructs—guided by the AF structural model (Figure 1A)—lacking the entire N-terminal PH-like domain (i.e., PH-like folds 1, 2, and 3: truncation of residues 1-347, termed DNAJC13_t347_, DNAJC13_t347(ylt1)_ and DNAJC13_t347(hpd)_).

These constructs expressed at similar levels with minimal proteolysis (Figures S6A,B). We found that removal of the DNAJC13 N-terminus (DNAJC13_t347_) blocked binding of DNAJC13 to PI(3)P *in vitro*, and that the J domain and C-terminal mutants did not rescue these phenotypes (Figures 4C-D, S6C). These findings were validated by live cell imaging and the GFP signal accumulation metric which showed that removal of the PH-like domain resulted in cytoplasmic DNAJC13—irrespective of the mutation status of the J domain or disordered C-terminus (Figures 4E,F). Thus, the enhancement in PI(3)P binding *in vitro* and endosomal localization in cells upon mutation of the DNAJC13 J domain or C-terminus has a complete dependence on the presence of a functional PH-like domain.

### PH-like domain requires oligomerization for efficient PI(3)P binding and endosomal localization

We next considered a possible mechanism by which relatively distal parts of the DNAJC13 protein could affect the function of its N-terminal PH-like domain. One of the known regulatory mechanisms for some proteins that bind PI(3)P is a requirement for multivalency. For example, the FYVE domains of EEA1, Hrs, and Frabin localize to endosomes poorly as isolated domains but localize efficiently when artificially oligomerized (Hayakawa et al., 2004). For EEA1, a stalk region upstream of the FYVE domain mediates dimerization between two monomers to position tandem FYVE domains for PI(3)P binding (Dumas et al., 2001). Additionally, recent studies of the *C. elegans* homolog RME-8 have proposed a model in which oligomerization of RME-8 is a critical part of its endosomal catalytic cycle (Norris et al., 2022). Thus, we wanted to determine if the PH-like domain of DNAJC13 was sufficient in isolation to localize to endosomes and bind PI(3)P or if, like a subset of other endosomal proteins, it required oligomerization.

**Figure 5.**
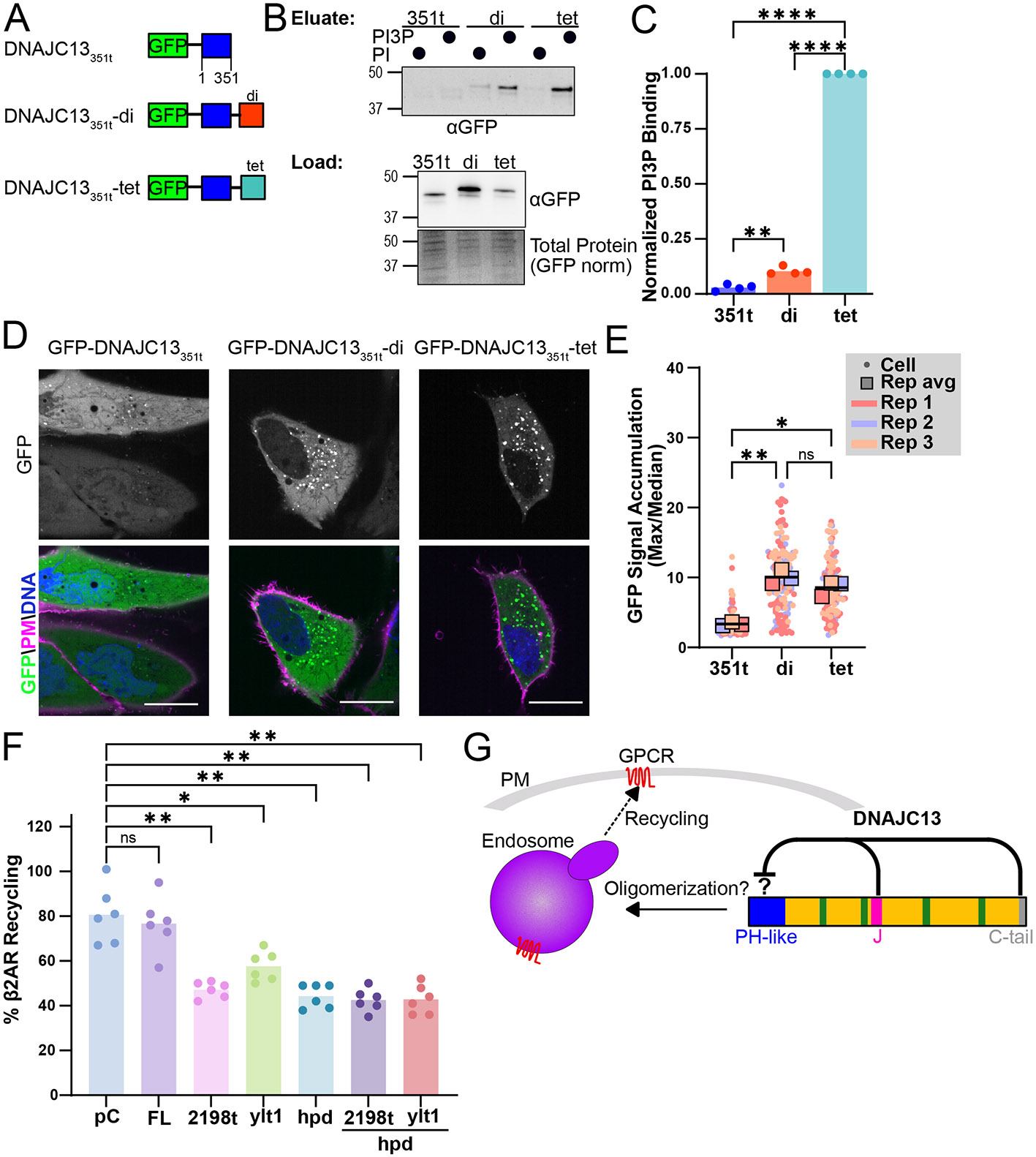
PH-like domain requires oligomerization for efficient PI(3)P binding and endosomal localization. ***A***, Domain schematics of GFP-tagged constructs containing only the PH-like domains (DNAJC13_351t_) and constructs containing exogenous dimerization (DNAJC13_351t_-dimer (di)) and tetramerization (DNAJC13_351t_-tetramer (tet)) motifs. ***B***, Western blots of PIP resin eluates for DNAJC13_351t_ constructs. GFP-DNAJC13_351t_ constructs were expressed in HEK293 cells and lysates, normalized by flow cytometry for GFP expression, and were bound to PI (control) and PI(3)P decorated agarose resins. Loads and eluates were run on SDS-PAGE (load total protein stain, bottom) and immunoblotted for anti-GFP (load, middle; eluate, top). ***C***, Quantification of PI(3)P pulldowns in B, normalized to load and the DNAJC13_351t_-tetramer pulldown (n=4 biological replicates, bar represents mean, one-way paired ANOVA with Tukey’s multiple comparisons corrections, p = 0.0025 (DNAJC13_351t_ vs DNAJC13_351t_-dimer), <0.0001 (DNAJC13_351t_ vs DNAJC13_351t_-tetramer), <0.0001 (DNAJC13_351t_-dimer vs DNAJC13_351t_-tetramer)). ***D***, Live cell spinning disk confocal microscopy images of GFP-DNAJC13_351t_ constructs in HeLa cells. Imaged with CellMask plasma membrane stain (magenta) and Hoechst DNA stain (blue) (scale bar = 20 μm), (representative example from n=3 biological replicates). ***E***, SuperPlot of cellular GFP signal accumulation metric of individual cells with single cell data shown in circles and biological replicate averages plotted in squares, colored by replicate. The black bars indicate the means of three biological replicate averages, with statistics performed on these averages (n=3 biological replicates, one-way paired ANOVA comparing biological replicate averages with Tukey’s multiple comparisons corrections, p=0.0088 (DNAJC13_351t_ vs DNAJC13_351t_-dimer), 0.0247 (DNAJC13_351t_ vs DNAJC13_351t_-tetramer), 0.0990 (DNAJC13_351t_-dimer vs DNAJC13_351t_-tetramer). **F**, β2AR recycling induced by 30 minutes isoproterenol (10 μM) treatment followed by 30 minutes alprenelol (10 μM) treatment measured 24 h after transfection with empty vector (pC) or DNAJC13 constructs. Cell surface receptor was measured by anti-FLAG-AF647 immunoreactivity and read out via flow cytometry in the far-red channel (n=6 biological replicates, one-way paired ANOVA comparing all DNAJC13 constructs against empty vector with Dunnett’s multiple comparisons corrections, p=ns (DNAJC13_FL_), 0.0027 (DNAJC13_2198t_), 0.0109 (DNAJC13_ylt1_), 0.0063 (DNAJC13_hpd_), 0.0061 (DNAJC13_2198t(hpd)_), 0.0061 (DNAJC13_ylt1(hpd)_). ***G***, Cartoon schematic of proposed mechanism whereby DNAJC13’s J domain and YLT motif in the C-terminal IDR inhibit oligomerization and localization to—and function on—endosomes in sorting pathways.

We designed constructs to express the N-terminal DNAJC13 PH-like domain, containing the three PH-like folds, in isolation (1-351, termed DNAJC13_351t_) and additionally made constructs fusing the PH-like domain to established dimerization and tetramerization motifs (DNAJC13_351t_-dimer and DNAJC13_351t_-tetramer, respectively) (Figure 5A) (Khairil Anuar et al., 2019). We first analyzed the binding of these constructs to PI(3)P beads in detergent lysate. Interestingly, we were unable to detect appreciable binding of the PH-like domain by itself to PI(3)P beads (Figures 5B-C, S7A). However, binding increased when the DNAJC13 PH-like domain was dimerized and was even further enhanced with tetramerization (Figures 5B-C, S7A). These observations demonstrate that like other PI(3)P-binding proteins, the DNAJC13 PH-like domain binds weakly to PI(3)P as a monomer and its binding is enhanced upon oligomerization.

To investigate the localization of the DNAJC13 PH-like domain in cells, we first confirmed the isolated, dimeric, and tetrameric constructs expressed at similar levels with minimal proteolysis (Figures S7B-C). By live cell microscopy, DNAJC13_351t_ looked similar to DNAJC13_FL_, with the GFP signal largely cytoplasmic and some vesicular localization (Figure 5D). Consistent with our *in vitro* assays, the dimerization or tetramerization of the DNAJC13PH-like domain enhanced its localization to vesicles which were confirmed to be endosomes with immunofluorescence imaging (Figures 5D, S7D). Using the GFP signal accumulation metric, we confirmed that DNAJC13_351t_-dimer and DNAJC13_351t_-tetramer had less homogenous distribution than the isolated DNAJC13 PH-like domain, consistent with their increased localization to endosomes (Figure 5E). We did not observe a difference in the degree of localization between the dimeric and tetrameric constructs, potentially due to saturation of PI(3)P binding sites in cells. Together, these data demonstrate that similar to other PI(3)P binding proteins, the DNAJC13 PH-like domain binds weakly to PI(3)P in isolation and its binding to PI(3)P—and therefore ability to localize to endosomes—can be enhanced by oligomerization.

### DNAJC13 mutants in the J domain or disordered C-terminus inhibits membrane protein recycling.

One function of DNAJC13 is to promote membrane protein recycling out of endosomes, and we have recently shown that knockdown of DNAJC13 inhibits recycling of a classical model GPCR, the β2AR (Girard et al., 2005; Popoff et al., 2009; Shi et al., 2009; Gomez-Lamarca et al., 2015b; Novy et al., 2024). As loss of DNAJC13 reduces β2AR recycling, we were interested to determine what happens in the context of increased DNAJC13 accumulation on endosomes such as what occurs upon overexpression of DNAJC13 carrying J domain or C-terminal mutants. We first examined DNAJC13_FL_ and found that overexpression of this construct had no effect on β2AR recycling relative to an empty vector control (Figure 5F). Comparatively, overexpression of DNAJC13 with single or double mutations to its J domain and/or disordered C-terminus resulted in an overt reduction in β2AR recycling (Figure 5F). Thus, DNAJC13 mutants which enhance its localization to endosomes can act in a dominant negative manner on β2AR recycling. Additionally, we observed a small but significant increase in agonist-induced β2AR internalization with overexpression of double DNAJC13 mutants and a trend toward increased β2AR internalization with DNAJC13_2198t_ and DNAJC13_hpd_ (Figure S7E). This is an often-observed phenotype following a large disruption of GPCR recycling and arises from a shift in the equilibrium between GPCR endocytosis and recycling during the 30-minute window of agonist-induced GPCR internalization (Tanowitz and von Zastrow, 2003; Lauffer et al., 2010; Dagunts et al., 2024). Together, these data demonstrate that mutants which increase DNAJC13 levels can act in a dominant negative manner on membrane protein recycling and suggest that tight control of DNACJ13 levels on endosomes may be critical to endosomal function.

## Discussion

Our findings demonstrate that DNAJC13 localization in cells is controlled by the cumulative function of three different domains: its N-terminal PH-like domain, which weakly binds PI(3)P, as well as its J domain and C-terminus, which act functionally upstream of the PH-like domain to oppose DNAJC13 localization to endosomes. Furthermore, we show that the poor endosomal localization of the DNAJC13 PH-like domain to endosomes can be improved by oligomerization, an observation consistent with a subset of other PI(3)P binding domains as well as recent findings that suggest the *C. elegans* homologue, RME-8, oligomerizes as part of its functional lifecycle (Klein et al., 1998; Dumas et al., 2001; Hayakawa et al., 2004; Norris et al., 2022). Functionally, we found that removing the negative regulation of DNAJC13 localization to endosomes resulted in dominant-negative phenotypes including an increase in endosomal clustering and reduction in endosomal recycling of a model cargo. Thus, in a working model we propose that DNAJC13 exists in an equilibrium between a cytoplasmic inhibited state and an oligomeric state that can localize efficiently to endosomes, with the transition between these states being controlled by a YLT motif in the disordered C-terminus and the catalytic triad, HPD, in the J domain (Figure 5G). We propose that the purpose for this multi-point regulation of DNAJC13 is to allow for precise control of DNAJC13 levels on endosomes such that endosomal cargo sorting is not disrupted by either too little or too much DNAJC13 activity.

### PI(3)P Binding Domains and Oligomerization.

Our data demonstrate that the isolated PH-like domain of DNAJC13 localizes poorly to endosomes in cells and weakly to PI(3)P *in vitro*, and this can be partially rescued through artificial oligomerization. This observation parallels what has been found for other PI(3)P binding domains like that from HRS, EEA1 and Frabin (Dumas et al., 2001; Hayakawa et al., 2004). For example, the PI(3)P binding domain in HRS associates with endosomes poorly as an isolated monomer but efficiently when artificially dimerized (Hayakawa et al., 2004). Multivalency in phosphoinositide binding is not limited to FYVE domains as a similar requirement has been shown for the PH-domain in dynamin (Klein et al., 1998; Lemmon, 2007). While not all PI(3)P binding proteins require oligomerization to bind to PI(3)P and endosomes (e.g., WDFY1 and endofin), multivalency—such as with EEA1—has been shown to allow for another layer of regulation (Blatner et al., 2004; Kim et al., 2005; Ramanathan and Ye, 2012). While oligomerization can assist PI(3)P binding in some cases, other proteins like DFCP1/ZFYVE1 have naturally occurring tandem FYVE domains that are required for high affinity PI(3)P binding (Cheung et al., 2001; Hayakawa et al., 2004). In this light it is interesting to note that a recent AF analysis of *C. elegans* RME-8 and human DNAJC13 revealed that the protein has a globular beta-sheet rich N-terminus that contains two additional PH-like folds (residues ~100-340) in addition to the PH-like fold (residues ~1-100) which had been previously identified and functionally validated (Norris et al., 2022). While our study did not attempt to disentangle the relative contributions of these three predicted PH-like folds in DNAJC13 binding to PI(3)P, it is notable that a previous study demonstrated that single point mutations (K17A, W20A, or Y24A) in the first of these PH-like folds were sufficient to block DNAJC13 binding to PI(3)P *in vitro* and endosomal localization in cells (Xhabija and Vacratsis, 2015). Thus, while future studies will be required to fully delineate roles for the second and third PH-like folds in DNAJC13 localization, the existing data from mutational analysis would suggest these second and third folds play structural roles rather than directly contributing to multivalency of PI(3)P binding; consistent with this model, our study showed that, in isolation, the three PH-like folds (1-351) bound weakly to PI(3)P *in vitro* and endosomes in cells and required oligomerization of multiple N-terminal domains to show detectable PI(3)P binding in our assays. Together, our study demonstrates that similar to a subset of other PI(3)P binding domains (Dumas et al., 2001; Hayakawa et al., 2004), the PI(3)P binding domain in DNAJC13 operates poorly in isolation and is enhanced by oligomerization.

The nature of our experiments allows for comparison of PI(3)P binding between the DNAJC13 PH-like domain (containing all three PH-like folds) in isolation, dimerized, and tetramerized, or in the full-length wild-type or mutationally activated, DNAJC13 constructs. One observation that arose from these comparisons is that full-length constructs bound to PI(3)P resins much better than the tetramerized PH-like domain (compare Figures S5A, S7A). This finding was interesting because, in cells, DNAJC13_FL_ and DNACJ13_351t_ showed a very similar phenotype. One potential explanation for this finding is that another PI(3)P binding protein functions cooperatively with DNAJC13 in binding PI(3)P resins; however, we consider this unlikely, as we and others have shown that deletion of the PH-like domain, or even a single point mutation in this domain, blocks binding of DNAJC13 to PI(3)P *in vitro* (Xhabija and Vacratsis, 2015). An alternate interpretation of our findings is that some of the negative regulation of DNAJC13_FL_ that occurs in cells is lost in detergent lysate, and thus full-length DNAJC13 spontaneously forms larger order assemblies (>4-mer) *in vitro* which enhance PI(3)P binding through multivalency. While future studies will be required to determine if DNAJC13 oligomerizes in cells, recent work on RME-8 identified a series of self-interactions which could allow for oligomerization (Norris et al., 2022). These interactions between RME-8 domains were first mapped by pulldown and yeast two-hybrid screens as occurring between the J domain and a C-terminal region of RME-8 (1650-2279), and the residues in the C-terminus were later mapped to D1657 and E1962 in repeating motifs called IWNs (Shi et al., 2009; Norris et al., 2017). While these C-terminal control points in RME-8 are different from those we identify in human DNAJC13, they point toward a general model of the DNAJC13/RME-8 C-terminus performing a regulatory role.

### DNAJC13 C-terminus as a Disordered Regulatory Region.

Intrinsically disordered regions often play regulatory roles in protein function (Fenton et al., 2023). Here we use two predictors of structural disorder, AF and JRonn, to demonstrate that the C-terminus of DNAJC13 is likely to be disordered. We then identified a novel and conserved motif we refer to as YLT1, consisting of Y2206, L2207, T2208, as a key negative regulator of DNAJC13 localization to endosomes in cells and ability to bind to PI(3)P *in vitro*. Broadly, we found that DNAJC13_ylt1_ phenotype was milder than that of DNAJC13_2198t_, with the DNAJC13_ylt1_ having a trend towards smaller effect on the GFP signal accumulation metric, β2AR recycling, and PI(3)P binding. This suggests the potential for additional determinants in the C-terminus that negatively regulate DNAJC13 localization to endosomes. Interestingly, we identify a second occurrence of the YLT sequence (YLT2; Y2215, L2216, T2217) downstream of YLT1, although we observed no overt effects upon mutation of YLT2. It is appealing to speculate that the YLT2 might be that additional determinant and that it can function cooperatively with YLT1 in control of DNAJC13 localization. Another feature of IDRs is that they are often the target of post-translational modification, and the C-terminus of DNAJC13 is in fact overrepresented in residues able to be phosphorylated (13 residues, 29% of residues) (Fenton et al., 2023). While future studies will be necessary to determine what the YLT1 motif interacts with, one potential model is that the DNAJC13 C-terminus makes autoinhibitory contacts within DNAJC13 itself, and that this interaction can be further regulated by dynamic phosphorylation and/or protein binding.

### J domain as a control point for DNAJC13 function.

While J domain-containing proteins are often thought of in terms of proteostasis, the role of J domains in membrane trafficking has been best studied in endocytosis where auxilin is involved in uncoating clathrin coated vesicles (Eisenberg and Greene, 2007). In this mechanism, auxilin binds clathrin, recruits HSC70, and stimulates the ATPase activity of HSC70 through the catalytic triad HPD in its J domain (Morgan et al., 2001; Eisenberg and Greene, 2007). The current model of DNAJC13/RME-8 function is that it recruits HSC70 to disassemble proteins on the endosomes including specific targets like clathrin and SNX1 (Girard et al., 2005; Popoff et al., 2009, 2009; Xhabija and Vacratsis, 2015). While it is worth noting that these experiments used loss of overall DNAJC13 as a proxy for J domain activity, similar effects on endosomal protein function were observed upon manipulation of HSC70 function (Zhang et al., 2001; Chang et al., 2004; Popoff et al., 2009; Shi et al., 2009). Our findings add to this model and demonstrate that disrupting the ability of DNAJC13 to interact with HSC70 also affects the levels of DNAJC13 on endosomes. Combined with our findings about an oligomerization requirement for DNAJC13 PH-like domain to associate with PI(3)P/endosomes, and the recent proposal that *C. elegans* RME-8 oligomerizes (Norris et al., 2022), it is intriguing to speculate that HSC70 regulates DNAJC13 localization and function through disassembly of DNAJC13 oligomers, thereby titrating DNAJC13 levels on endosomes by controlling the functional affinity of DNAJC13 toward PI(3)P.

### Other regulators of DNAJC13 localization to endosomes.

In our study we took advantage of the largely cytoplasmic phenotype GFP-DNAJC13_FL_ to perform a structure-function analysis of domains in DNAJC13 which negatively regulate its localization to endosomes. However, it is interesting to note that other groups—working with a similar construct but in different cell types—saw a variation in phenotypes including a mostly cytoplasmic GFP-DNAJC13_FL_ to mostly localized GFP-DNAJC13_FL_ (Fujibayashi et al., 2008; Freeman et al., 2014; Xhabija and Vacratsis, 2015). While one possible explanation of these differences is a cell type dependent component such as differential protein expression, it is notable that one study that identified GFP-DNAJC13_FL_ as primarily localized to endosomes used a pre-fixation digitonin treatment (Fujibayashi et al., 2008), which specifically reduces cytoplasmic signal (Liu et al., 2001). While we focus here on overexpressed DNAJC13, multiple groups—including our own—have shown that endogenous DNAJC13/RME-8 is primarily localized to endosomes.(Zhang et al., 2001; Fujibayashi et al., 2008; Freeman et al., 2014; Novy et al., 2024). This observation raises the question as to what other regulators—in addition to the N-terminus, J domain, and C-terminus—may control DNAJC13 localization in cells. One interesting possibility is that while DNAJC13 localization to endosomes requires N-terminal PH-like domain binding to PI(3)P, it is possible that interactions with other binding partners (e.g. SNX1, FAM21) which–while not necessary for its endosomal localization–may help stabilize DNAJC13 on endosomes (Harbour et al., 2012; Jia et al., 2012; Helfer et al., 2013; Freeman et al., 2014; Xhabija and Vacratsis, 2015; Dostál et al., 2023).

### Dominant Negative Activity of DNAJC13 J domain and C-terminal mutants.

We observed that mutation of DNAJC13 J domain, or disordered C-terminus, removed negative regulation controlling binding of its PH-like domain to PI(3)P and resulted in increased localization of DNAJC13 to endosomes in cells. Intriguingly, we observed that overexpression of DNAJC13 carrying these mutations had dominant negative effects on endosomes and caused increased endosomal perinuclear clustering and a reduction in recycling of a model GPCR, the β2AR. Future studies will be required to determine the mechanism(s) by which these DNAJC13 mutants produce dominant negative activity at endosomes; while one model would be that increased DNAJC13 levels on endosomes results in an excess DNAJC13 activity, it is also possible that these DNAJC13 mutants are competing with the endogenous DNAJC13 for key proteins partners including SNX1 or FAM21 (Shi et al., 2009; Freeman et al., 2014). While the mechanism by which these DNAJC13 mutants cause perinuclear endosomal clustering and reduced β2AR recycling is unclear, it is intriguing to note that perinuclear endosomal clustering/collapse was observed upon loss of function of two DNAJC13 functional partners: the WASH complex and clathrin heavy chain. Specifically, loss of WASH complex function (knockout of the WASH1 subunit) or disruption of clathrin function (overexpression of the dominant negative hub domain of clathrin heavy chain) results in a redistribution of EEA1 positive endosomes from distributed throughout the cell to tightly clustered/collapsed near the nucleus (Bennett et al., 2001; Gomez et al., 2012). Thus, it is possible that all three manipulations—overexpression of DNAJC13 mutants, WASH1 knockout, or dominant negative clathrin—share a common mechanistic basis for endosomal clustering, possibly by disrupting the connection between the WASH complex and the dynein/microtubule system, which promotes endosomal translocation to the perinuclear region (Fokin and Gautreau, 2021; Fokin et al., 2021).

Together, our study examined how human DNAJC13, a protein important in endosomal sorting, is regulated. We identify that DNAJC13 localization to endosomes is controlled by the low affinity of its PH-like domain for PI(3)P, which can be overcome by oligomerization, and the negative regulation promoted by its J domain and disordered C-terminus. Future studies will be important in showing how these novel control points integrate cellular signals to tune DNAJC13 function on endosomes and thereby control efficient cargo sorting into the recycling and degradative pathways.

## Materials and Methods

### Chemicals

From Corning, Dulbeco’s Phosphate Buffered Saline (DPBS) DPBS with (21-030-CM) or without (21-031-CV) Calcium and Magnesium. Bovine Serum Albumin (Sigma Aldrich, A7030) was dissolved in DPBS with Calcium and Magnesium and filtered before use. For cell fixation for microscopy, 16% paraformaldehyde ampules were purchased from Invitrogen (Thermo Scientific, 28906) and diluted to 4% in DPBS with Calcium and Magnesium immediately before use.

### Antibodies

All antibodies used are commercially available and validated by the vendor, no additional validation was performed in lab. From Cell Signaling, mouse anti-EEA1 (Cell Signaling, 48453S) and mouse anti-GFP (55494S), rabbit anti-GM130 (Cell Signaling, 12480T, for imaging). From Novus Biologicals, rabbit anti-GFP (Novus Biologicals, NB600-308, for imaging). From Takara Biosciences, mouse anti-GFP (Clontech Labs 3P 632381, for western blot). Secondary antibodies for imaging from Invitrogen – goat anti-mouse AF488 (Thermo Scientific, A11029), goat anti-rabbit AF488 (Thermo Scientific, A32731), Goat anti-mouse AF647 (Thermo Scientific, A21235), goat anti-rabbit AF647 (A32733). Secondary antibodies for western blotting from Bio Rad goat anti-mouse StarBrite 700 (Bio-Rad, 12004158).

### Structural prediction

The AlphaFold2 structural prediction was downloaded from the AlphaFold Protein Structural Database (https://alphafold.ebi.ac.uk/entry/O75165) (Varadi et al., 2022) and visualized in Pymol. For AlphaFold3 structural prediction, the sequence for human DNAJC13 (Uniprot O75165) was input into the DeepMind AlphaFold3 server (https://golgi.sandbox.google.com/) with a random seed (Abramson et al., 2024). All models were downloaded and viewed separately in Pymol, where the final 73 residues were each given a score of 1 for unstructured and 0 for structured. The average of the 5 models is shown in Figure S1A.

For JRonn disorder prediction, the sequence for DNAJC13 (O75165) was opened in Jalview (Waterhouse et al., 2009) and the C-terminal 257 amino acids were run through the homology-based secondary structure JPred algorithms, including the JRonn disorder predictor algorithm.

### Sequence conservation

To assess the C-terminus for sequence conservation, all vertebrate (plus *D. melanogaster* and *C. elegans*) orthologues for human DNAJC13 were downloaded from the Ensembl database (Harrison et al., 2024) as a multiple sequence alignment. This alignment was opened in Jalview, trimmed to show only sequences aligning with the human C-terminus and relative conservation score was calculated (Waterhouse et al., 2009).

### DNA constructs

All plasmids were verified either via Sanger sequencing of several reads or whole plasmid nanopore sequencing. pcDNA3-SSF-β2AR was a gift from M. bon Zastrow (UCSF). pEGFP-DNAJC13 was a gift from the Sekiguchi group(Fujibayashi et al., 2008). Upon sequencing of our construct, we noticed a nonnative sequence on the C-terminus (HRPLPGSTGSR) and removed this sequence by re-cloning the native sequence into the parental pEGFP-C1 vector between restriction sites KpnI and BamHI and the resulting construct is what we refer to as DNAJC13_FL_. To create the C-terminally tagged DNAJC13_FL_, GFP was PCR amplified and inserted to the C-terminus of pEGFP-DNAJC13_FL_ using NEBuilder (New England Biologicals, E2621L) to insert at the BamHI site. After successful insertion, the N-terminal GFP was removed by digestion with AgeI and KpnI, and NEBuilder to stitch the plasmid back together with a new start codon, creating pEGFP-DNAJC13_FL_-ctGFP. This construct begins with the linker between the original N-terminal GFP and DNAJC13 (GGGSGGGS).

PCR, digestion and ligation with KpnI and BamHI were again used to copy specific regions and re-insert into the parental pEGFP-C1 vector for truncated protein DNAJC13_2198t_ from DNAJC13_FL_. To perform the alanine scanning of the C-terminus, double stranded gBlocks from IDT were obtained containing the mutant sequences as well as homology arms for assembly with NEBuilder after digestion of pEGFP-DNAJC13_2198t_. To mutate the DnaJ domain residues (HPD) to alanine, a shorter construct encoding residues 1-1927 of DNAJC13 was cloned into pEGFP-C1 vector between KpnI and BamHI. Next, a gBlock from IDT was obtained encoding for a fragment of DNAJC13 with the HPD residues mutated to alanine and inserted between internal cut sites BlpI and PshAI with NEBuilder. Next, the C-terminus encoding 1927-2198 or 1927-end was copied via PCR and inserted into the end of the truncated, hpd mutant construct after the BamHI site using NEBuilder, creating DNAJC13_hpd_ and DNAJC13_2198t(hpd)_.

Truncated proteins DNAJC13_t347_, DNAJC13_t347(ylt1)_, DNAJC13_t347(hpd)_, and DNAJC13_351t_ were created by PCR of the region from DNAJC13_FL_, or DNAJC13_ylt1_ or DNAJC13_hpd_ for the respective mutants, and reinsertion (via NEBuilder for DNAJC13_t347_ constructs, and classical linear ligation for DNAJC13_351t_) into the parental pEGFP-C1 vector between KpnI and BamHI. To add dimerizing and tetramerizing domains to 351t, dimerizing and tetramerizing motifs were codon corrected from the original sequence for bacterial expression (Khairil Anuar et al., 2019) for human cell expression and ordered as gBlocks from IDT with homology overlaps for cloning into pEGFP-DNAJC13_351t_ at the BamHI site.

### Cell culture

FLP-In-293 (Thermo Scientific, R75007) cells were purchased from Thermo Fisher Scientific, and HeLa (ATCC, CCL-2) and HEK293 (CRL-1583, ATCC) were purchased from ATCC. For β2AR experiments, HEK293 cells were engineered to stably express CMV-β2AR, maintained with addition of 1:1000 G418 (Geneticin, Thermo Fisher Scientific 10131035). All cells were grown in DMEM (Thermo Fisher Scientific, 11965-092) supplemented with 10% FBS, at 37°C and 5% CO2. Cell lines were not further authenticated after receiving from the vendor. Testing for Mycoplasma contamination was performed every ~6 months on all cell lines.

### Plasmid transfection

For microscopy, flow cytometry, and western blot experiments, HeLa cells were plated at 50% confluence in dishes for the respective experiment. The next day they were transfected using Lipofectamine-2000 (Thermo Scientific, 11668019) and OptiMEM (Gibco, 31985088). DNA, lipofectamine, and OptiMEM was scaled for the experiment and DNA/lipofectamine-200 used depended on the length of the construct, with bigger constructs having more DNA/lipofectamine and smaller constructs less. DNAJC13_FL_, DNAJC13_hpd_, and triplet scanning mutants were all transfected at 1.25x amounts, while DNAJC13_2198t_, DNAJC13_2198t(hpd)_, DNAJC13_t347_, DNAJC13_t347(ylt1)_, and DNAJC13_t347(hpd)_ were transfected at 1x amounts, and DNAJC13_351t_, DNAJC13_351t_-dimer and DNAJC13_351t_-tetramer were transfected at .75x amounts.

Cells for imaging experiments were grown in 8 well imaging dishes (Thermo Scientific, 155409) were transfected with Lipofectamine-2000 (0.643 μL 1x) and DNA (300 ng 1x) in OptiMEM (50 μL). Cells for flow cytometry experiments were grown in 12 well dishes and were transfected with Lipofectamine-2000 (1.875 μL 1x) and DNA (875 ng 1x) in OptiMEM (400 μL). Cells for western blot expression experiments were grown in 6 well dishes and were transfected with Lipofectamine-2000 (5.14 μL 1x) and DNA (2400 ng 1x) in OptiMEM (400 μL). Fixed microscopy experiments were performed in 24 well dishes containing #1.5 thickness round cover slips (Harvard Apparatus, 64-0712) coated in 1:100 Poly-L-Lysine (Sigma-Aldrich, P8920-100ML) and were transfected with Lipofectamine-2000 (1.22 μL 1x) and DNA (570 ng 1x) in OptiMEM (120 μL).

For PIP binding studies, FLP-In-293 cells were used instead of HeLa cells. They were plated at 40% confluence in T25s, the next day they were transfected with Lipofectamine-2000 (27.3 μL) and DNA (13.3 μg) in OptiMEM (1 mL). For β2AR recycling assays, HEK293 cells stably expressing SSF-B2AR were seeded at 50% confluence in 6 well plates. The next day they were transfected with Lipofectamine-2000 (10 μL) and DNA (5 μg) in OptiMEM (300 μL).

### Flow cytometry for expression

One day after transfection with GFP-DNAJC13 constructs, cells were washed with DPBS without Ca/Mg and lifted in TrypLE (Gibco, 12604021) and resuspended in Flow Buffer (DPBS+Ca/Mg + 1% BSA). Cells were analyzed using a Beckman Coulter CytoflexS. For each experiment, 10,000 counts were taken after discrimination of cells (forward vs side scatter) and singlets (forward scatter vs forward scatter width). Data was then reanalyzed via FlowJo to gate for cells and singlets and assess the geometric mean of the FITC-A channel (488 nm laser, 525/40 nm filter).

### Live cell microscopy

One day after transfection with GFP-DNAJC13 constructs, cells were treated with 1:4000 Invitrogen CellMask Deep Red Plasma membrane stain (Thermo Scientific, C10046) and 1:500 Pierce Hoechst-33342 DNA stain (Thermo Scientific, 62249) diluted in pre-equilibrated Fluorobrite (Thermo Scientific, A1896701). After 10 minutes in the incubator, media was replaced with fresh, pre-equilibrated Fluorobrite and moved to the imaging incubator (35°C) on a Nikon spinning disk confocal microscope (Yokogawa CSU-W1 on a Nikon TiE). Cells were imaged under a 100x oil immersion objective (1.49 NA, Apochromat TIRF, 12 mm working distance) with the blue channel (405 nm laser, 445/50 nm filter), green channel (488 nm laser, 525/36 nm filter), and far-red channel (640 nm, 700/75 nm filter). Each construct was imaged over three biological replicates, taking 6-12 images per construct each replicate.

### Blinded analysis of phenotype

All images were collected in an unblinded manner cells were manually sectioned, with regions of interest (ROIs) drawn by hand and saved in FIJI-ImageJ. Investigators were then blinded when assessing the phenotype. All healthy, expressing cells were included for analysis. Images and ROI sets for all constructs to be blinded (GFP-DNAJC13_FL_ and GFP-DNAJC13_2198t_ for figure 1; GFP-DNAJC13_FL_ and GFP-DNAJC13_ylt1_ for figure 2; GFP-DNAJC13_2198t_, GFP-DNAJC13_hpd_, GFP-DNAJC13_2198t(hpd)_, and GFP-DNAJC13_ylt1+hpd_ for figure 3) were renamed to randomized numbers. Individual cells were scored into two phenotypes for figure 1 and 2 as follows: “cytoplasmic” if GFP predominantly localized to the cytoplasm in addition to localized puncta; and “localized” if GFP was predominantly localized to puncta with little to no cytoplasmic signal. In separate blinding and scoring of new images for figure 3: “cytoplasmic” if GFP predominantly localized to the cytoplasm in addition to localized puncta; “distributed” if GFP-positive puncta were spread across the cell with only dim cytoplasmic signal; and “clustered” if GFP-positive puncta were largely confined to three or fewer contiguous structures with only dim cytoplasmic signal.

### GFP signal accumulation metric

Cells were manually sectioned and analyzed for maximal and median pixel intensity of the green channel in FIJI-ImageJ. For samples that had blinded phenotypic analysis performed, ROIs were the same ones used in both analyses to allow for direct comparison of phenotype and quantitative metrics. GFP signal accumulation was found by dividing the maximal pixel intensity by the median pixel intensity. All healthy, expressing cells imaged over the three biological replicates were included as individual points for analysis, and the mean scores from each biological replicate were compared in statistical analysis as a SuperPlot.

### Fixed microscopy

One day after transfection, coverslips were washed with DPBS+Ca/Mg before fixing for 20 minutes with 4% paraformaldehyde while rocking at RT. Cells were rinsed 3x with DPBS+Ca/Mg, blocked and permeabilized for 30 minutes, rocking at RT with Imaging Block Buffer (DPBS+Ca/Mg+4% BSA+0.1%TritonX), then incubated with primary antibodies overnight, rocking at 4°C (1:1000 rabbit anti-GFP and 1:500 mouse anti-EEA1, or 1:1000 mouse anti-GFP (Cell Signaling) and 1:1000 rabbit anti-GM130, diluted in Imaging Block Buffer). The next day, cover slips were rinsed 3x with DPBS+Ca/Mg, incubated with secondary antibodies (1:2000 anti-Mouse-488 & anti-Rabbit-647 or 1:2000 anti-Rabbit-488 & anti-Mouse-647 in Imaging Block Buffer) for 1 hour rocking at RT before being washed 3x with DPBS+Ca/Mg and mounted on fresh glass slides with ProLong Diamond + DAPI (Thermo Scientific, P36962).

At least one day after mounting, cells were imaged using the same Nikon spinning disk confocal microscope used for live microscopy. On three separate biological replicates for all constructs analyzed with fixed microscopy, 5 fields of view were imaged with Z-stacks covering whole cells. A representative example of a single z-plane is shown.

### SDS-PAGE sample preparation

For analyzing expression of GFP-tagged constructs, one day after transfection, cells were washed once with DPBS and lifted with TrypLE. Cell pellets were collected and lysed on ice for 10 minutes with 250 μL RIPA Buffer (50 mM Tris pH 7.4, 150 mM NaCl, 1% TritonX, 0.5% sodium deoxycholate, 0.1% sodium dodecyl sulfate) with HALT protease inhibitor cocktail (Thermo Scientific, 78430). Cells were further lysed via sonication (1s on/3 s off, 3 cycles at 35% amplitude). Lysates were then clarified at 10,000 x g for 10 minutes at 5°C and a sample was combined with 4x SDS PAGE Sample Buffer (250 mM Tris, pH 6.8, 40% glycerol, 8% SDS, bromophenol blue) + beta-mercaptoethanol and heated at 95°C for 5 minutes.

### Western blotting protocol

Samples were loaded along with ladder (Bio-Rad; 1610363, 1610373, 1610377; or GoldBio, P007) onto gradient Bio-Rad 4-20% polyacrylamide SDS-PAGE gels containing StainFree total protein stain (Bio-Rad, 456-8095) and run at 125V in SDS-PAGE running buffer (250.1 mM Tris, 1.924 M glycine, 0.0347 M SDS) until dye front ran off the gel. StainFree total protein stain was activated on a Bio-Rad ChemIDoc Imaging System and imaged before transfer onto nitrocellulose with the Bio-Rad TurboBlot Transfer system (Bio-Rad, 1704150). Blots were then blocked in Bio-Rad EveryBlot Blocking Buffer (Bio-Rad, 12010020) for ~90 min rocking at RT, then primary antibody (Takara Biosciences mouse-anti-GFP, 1:1000) was diluted in Western Blot Antibody Buffer (1xTBS pH 7.4 + 5% BSA + 0.1% TritonX) and rocked at 4°C overnight. Blots were washed four times with PBST (DPBS+0.1%TritonX). Bio-Rad StarBrite secondary antibody (1:3000, diluted in PBST) were incubated for 1 hour rocking at RT before being washed four times with PBST and imaged on the Bio-Rad ChemIDoc.

### β2AR recycling assay

Three wells were transfected per construct. The day after transfection, in one well media was replaced with equilibrated media containing 10 μM isoproterenol. Cells were treated for 30 minutes before rinsing once and replacing with 10 μM alprenelol and treated for another 30 minutes (Recycled well). The other wells were treated for 30 minutes with either just 10 μM isoproterenol for 30 minutes (Internalized) or 10 μM alprenelol for 30 minutes (Total). After agonist and/or antagonist treatment, cells were rinsed and lifted with TrypLE before resuspending with Flow Buffer with 1:2000 M1(anti-FLAG)-647 for surface labeling of receptor. Cells were labeled on rotisserie for ~1 hour before resuspending in Flow Buffer and were analyzed on a BD Symphony, analyzing 10,000 cells after gating for cells, singlets, and GFP-DNAJC13 expression (561 nm laser, 525/40 nm emission filter) as assessed against a nonexpressing control. Data was re-analyzed after collection on FlowJo and the population geometric mean for the far-red, AF647 channel (638 nm laser, 660/20 nm emission filter). Internalization was defined by 1-Internalized/Total and recycling was defined by (Recycled-Internalized)/(Total-Internalized).

### Phosphatidylinositol phosphate (PIP) binding studies

Protocols adapted from (Xhabija and Vacratsis, 2015). In brief, HEK293 cells were seeded in a T25 at 40% confluence. 24 hours later, they were transfected with GFP-DNAJC13 constructs. The next day, cells were lifted with TrypLE, quenched with DMEM, a small sample was resuspended in Flow Buffer and analyzed on a Beckman Coulter Cytoflex S (see Flow Cytometry for expression). Using FITC-A geometric mean to normalize GFP loading, cells were lysed in a varying amount of PIP Lysis Buffer (50 mM Tris, pH 7.4, 76 mM NaCl, 1% TritonX, 10% glycerol, 2 mM EGTA) with HALT protease inhibitor cocktail, on ice by sonication (1s on/3 s off, 7 cycles @35% amplitude). A portion of lysate was then clarified by centrifugation (15,000 x g, 10 min, 4°C). A sample of clarified lysate was taken for western blot analysis and 250 μL loaded onto phosphatidylinositol or phosphoinositide decorated resins (50 μL slurry) - PI (Echelon Biosciences, P-B001) and PI(3)P (Echelon Biosciences, P-B003A), pre-equilibrated in PIP Lysis Buffer. Lysates were bound for 2 hours on a rotisserie at 4°C. Resins were then washed three times in PIP Wash Buffer (10 mM HEPES pH 7.4, 150 mM NaCl, 0.25% TritonX) before elution with 2xSDS PAGE Sample Buffer (diluted from 4x in PIP Wash Buffer) at 70°C for 10 minutes.

### Statistical analysis and reproducibility

Statistical analysis was performed in Prism (GraphPad). Sample sizes for experiments were predetermined based on field standard practices and previous experience in the lab with a particular technique. All experiments come from at least three biological replicates. No data was excluded from analysis. As all data came from six or fewer biological replicates, normality tests were not performed. Plotted microscopy data are represented as individual biological replicates, or as SuperPlots with the means of three biological replicates, as well as data from individual cells across replicates, where replicate averages were compared for statistical analysis (Lord et al., 2020). Expression western blots were performed on three separate experiments for all constructs and a representative example is shown. All measurements were taken from distinct samples, except as follows: Flow cytometry data for expression of DNAJC13_FL_ and DNAJC13_2189t_ is reused between 1B, S3B (all data was collected at one time). Statistical test performed is noted in each figure legend. Tests were performed as appropriate; paired two-tailed t-test, paired one-way ANOVA followed by Dunnett’s multiple comparisons corrections, paired one-way ANOVA followed by Tukey’s multiple comparisons corrections, or unpaired one-way ANOVA followed by Dunnett’s multiple comparisons corrections. P values are represented as follows: ns if P>0.05, * if P<= 0.05, ** if P <= 0.01, *** if P <= 0.001, and **** if P <= 0.0001.

### Software and code

Data were collected with the following software: flow cytometry (Beckman CytExpert, v2.4), western blot (Bio-Rad Image Lab Touch v2.4.0.03 and FIJI-ImageJ v2.14.0/1.54f), and microscopy (Nikon Elements v4.51.01 (Build 1146)). Data were analyzed with the following software: statistical analysis and graphing (GraphPad Prism v10.3.1), flow cytometry (FlowJo v10.10.0), and microscopy (FIJI-ImageJ v2.14.0/1.54f). JRonn modeling and conservation analysis were performed in Jalview (v 2.11.4.1). Structural analysis of models was performed in Pymol (Schrodinger Pymol v 2.5.7).

## Supplementary Material

Figure S1 **Figure S1. *A***, Structural prediction for the C-terminus of DNAJC13 (sequence, above), with JRonn disorder prediction (middle) and summary from five AlphaFold3 structural predictions (bottom). ***B***, Uncropped anti-GFP western blot (left) and total protein stain gel (right) from Figure 1C; cropped area shown in the black box. ***C***, Western blot (anti-GFP, left) and total protein stain (right) of extracts from three replicates of HeLa cells transfected with DNAJC13_FL_ (at a 1:10 dilution of a standard load) or DNAJC13_FL_-ctGFP (undiluted), and a nontransfected control (Control). The white arrowhead points to DNAJC13. ***D***, Fixed immunofluorescence microscopy image of GFP-DNAJC13_FL_ expressed in HeLa cells. Imaged with anti-GFP (green), endosomal marker EEA1 (magenta), and DAPI DNA stain (blue) with insets shown to the right (scale bar = 20 μm, 5 μm in inset), (representative example from n=3 biological replicates). A line-scan (yellow line) showing normalized fluorescence intensity of GFP (green) and EEA1 (magenta) signal are plotted along the line (right). ***E***, Cross-comparison of two phenotype assays, GFP signal accumulation metric data from Figure 1F, color coded by blinded phenotype analysis from Figure 1G.

Figure S3 **Figure S3. *A***, Representative western blot (anti-GFP, left) and total protein stain gel (right) of extracts from HeLa cells transfected with DNAJC13_FL_, DNAJC13_2198t_, DNAJC13_hpd_, or DNAJC13_2198t(hpd)_ and a nontransfected control (Control) in HeLa cells (n=3 biological replicates). The arrowhead marks GFP-DNAJC13, and the # marks free GFP. ***B***, Representative western blot (anti-GFP, left) and total protein stain gel (right) of HeLa cells transfected with DNAJC13_FL_, DNAJC13_ylt1(hpd)_ and a nontransfected control (Control) in HeLa cells (n=3 biological replicates). The arrowhead marks GFP-DNAJC13, and the # marks free GFP. ***C***, Flow cytometry-based expression analysis of DNAJC13_FL_, DNAJC13_2198t_, DNAJC13_hpd_, and DNAJC13_2198t(hpd)_ in HeLa cells, assessed by geometric mean of GFP channel and displayed as fold above background signal from untransfected cells (n=3 biological replicates, bar represents mean). DNAJC13_FL_ and DNAJC13_2198t_ data is the same as appears in 1B, all data acquired at same time. ***D***, Flow cytometry-based expression analysis of DNAJC13_FL_ or DNAJC13_ylt1(hpd)_ in HeLa cells, assessed by geometric mean of GFP channel and displayed as fold above background signal from untransfected cells (n=3 biological replicates, bar represents mean).

Figure S2 **Figure S2. *A***, Representative western blot (anti-GFP, left) and total protein stain gel (right) of extracts from HeLa cells transfected with DNAJC13_FL_ or triplet scanning mutants, and a nontransfected control (Control) (n=3 biological replicates). The arrowhead marks GFP-DNAJC13 and the # marks free GFP. ***B***, Flow cytometry-based expression analysis of constructs expressed in HeLa cells, assessed by geometric mean of GFP channel and displayed as fold above background signal from untransfected cells (n=3 biological replicates, bar represents mean). ***C***, Fixed immunofluorescence microscopy image of GFP-DNAJC13_ylt1_ expressed in HeLa cells. Imaged with anti-GFP (Green), Golgi marker anti-GM130 (magenta), and DAPI DNA stain (blue) with insets shown to the right (scale bar = 20 μm, 5 μm in inset), (representative example from n=3 biological replicates). A line-scan (yellow line) showing normalized fluorescence intensity of GFP (green) and GM130 (magenta) signal are plotted along the line (right). ***D***, Cross-comparison of two phenotype assays, GFP signal accumulation metric data from Figure 2D, color coded by blinded phenotype analysis from Figure 2E. ***E***, GFP signal accumulation metric data for DNAJC13_FL_ and DNAJC13_ylt1_, previously shown in Figure 2D, with signal to noise cutoffs reveals lower population of DNAJC13_ylt1_-expressing cells by GFP signal accumulation scores are often low expressing cells. Numbers above datasets represent total number of cells in the dataset, red bars indicate the mean of the overall dataset. ***F***, Representative images of DNAJC13_FL_ and DNAJC13_ylt1_ containing high- and low-expressing cells in the same field of view. Cellular ROIs are drawn, with the GFP signal accumulation score annotated in white on the image (scale bar = 20 μm). In each image, a single non-expressing cell is analyzed, as annotated with the yellow line.

Figure S4 **Figure S4. A**, Live cell spinning disk confocal microscopy images of GFP-DNAJC13_2198t(hpd)_ in HeLa cells showing distributed (left) and clustered (right) endosomes. Imaged with CellMask plasma membrane stain (magenta) and Hoechst DNA stain (blue) (scale bar = 20 μm) (phenotypic representative examples from n=3 biological replicates). ***B***, Fixed immunofluorescence microscopy image of GFP-DNAJC13_2198t(hpd)_ expressed in HeLa cells. Imaged with anti-GFP (Green), DAPI DNA stain (blue), and endosomal marker anti-EEA1 (magenta, top) or Golgi marker anti-GM130 (magenta, bottom). Insets shown to the right (scale bar = 20 μm, 5 μm in inset), (representative example from n=3 biological replicates). Line-scans (yellow lines) showing normalized fluorescence intensity of GFP (green) and EEA1 (magenta) or GM130 (magenta) signal are plotted along the lines (right). **C**, Live cell spinning disk confocal microscopy images of GFP-DNAJC13_ylt1(hpd)_ in HeLa cells showing distributed (left) and clustered (right) endosomes. Imaged with CellMask plasma membrane stain (magenta) and Hoechst DNA stain (blue) (scale bar = 20 μm) (phenotypic representative examples from n=3 biological replicates). ***D***, GFP signal accumulation metric data for DNAJC13_2198t(hpd)_, previously shown in Figure 3C, with signal to noise cutoffs for exclusion of data reveals one replicate with low signal to noise cells. Individual cells are shown as circles, with replicate averages shown in squares, data color coded by replicate, with black bars representing the average of the three biological replicate averages. **E**, Cross-comparison of two phenotype assays, GFP signal accumulation metric data from Figure 3C, color coded by blinded phenotype analysis from Figure 3D.

Figure S5 **Figure S5. *A***, Uncropped blots (anti-GFP) and total protein stain gel from Figure 4A; cropped areas shown in the black boxes. Extra lanes are from a different experiment (see 5B/S7A). **B**, Western blots of PIP resin eluates for DNAJC13_FL_ and double mutant GFP-DNAJC13_ylt1(hpd)_. GFP-DNAJC13_FL_ and GFP-DNAJC13_ylt1(hpd)_ were expressed in HEK293 cells and lysates, normalized by flow cytometry for GFP expression, and were bound to PI (control) and PI(3)P decorated agarose resins. Loads and eluates were run on SDS-PAGE (load total protein stain, bottom) and immunoblotted for anti-GFP (load, middle; eluate, top). ***C***, Uncropped blots (anti-GFP) and total protein stain gel from B. **D**, Quantification of PI(3)P pulldowns in B/C, normalized to load and the full-length pulldown (n=4 biological replicates, bar represents mean, paired two-tailed t-test, p=0.0481).

Figure S6 **Figure S6. A**, Representative western blot (anti-GFP, left) and total protein stain gel (right) of extracts from HeLa cells transfected with DNAJC13_FL_, DNAJC13_t347_, DNAJC13_t347(ylt1)_, or DNAJC13_t347(hpd)_, and a nontransfected control (Control) (n=3 biological replicates). Arrowhead marks GFP-DNAJC13 and the # marks free GFP. ***B***, Flow cytometry-based expression analysis of t347 constructs in HeLa cells, assessed by geometric mean of GFP channel, displayed as fold above background signal from untransfected cells (n=3 biological replicates, bar represents mean). ***C***, Uncropped blot (anti-GFP) and total protein stain gel from Figure 4C, cropped area shown in the black boxes.

Figure S7 **Figure S7. *A***, Uncropped blots (anti-GFP) and total protein stain gel from Figure 5B – gel was run with samples from Figure 4A, image was re-thresholded for viewing relevant samples, with cropped area shown in the black (or white) box. ***B***, Flow cytometry-based expression analysis of DNAJC13_351t_ constructs in HeLa cells, assessed by geometric mean of GFP channel, displayed as fold above background signal from untransfected cells (n=3 biological replicates, bar represents mean). ***C***, Representative western blot (anti-GFP, left) and total protein stain gel (right) of extracts from HeLa cells transfected with DNAJC13_351t_, DNAJC13_351t_-dimer, or DNAJC13_351t_-tetramer, and a nontransfected control (Control) (n=3 biological replicates). Arrowhead marks GFP-DNAJC13_FL_, double arrowhead marks GPF-DNAJC13_351t_ and the # marks free GFP. ***D***, Fixed immunofluorescence microscopy image of GFP-DNAJC13_351t_-tetramer expressed in HeLa cells. Imaged with anti-GFP (Green), DAPI DNA stain (blue), and endosomal marker anti-EEA1 (magenta, left) or Golgi marker GM130 (magenta, right) with insets shown to the right (scale bar = 20 μm, 5 μm in inset), (representative example from n=3 biological replicates). Line-scans (yellow lines) showing normalized fluorescence intensity of GFP (green) and EEA1 (magenta) or GM130 (magenta) signal are plotted along the line (right). ***E***, β2AR internalization induced by 30 minutes isoproterenol (10 μM) treatment measured 24 h after transfection with empty vector (pC) or DNAJC13 constructs. Cell surface receptor was measured by anti-FLAG-AF647 immunoreactivity and read out via flow cytometry in the APC channel (n=6 biological replicates, one-way paired ANOVA comparing all DNAJC13 constructs against empty vector with Dunnett’s multiple comparisons corrections, p= 0.0064 (DNAJC13_2198t(hpd)_), 0.0229 (DNAJC13_ylt1(hpd)_), ns for all other comparisons.

This article contains supporting information.

## Data Availability

All data generated and analyzed in this study are included as figures or supplementary information.

## Significance Statement

- The mechanisms which control DNAJC13 recruitment to endosomes are not fully understood.
- Here the authors show that endosomal localization of DNAJC13 is regulated through the coordinated action of three of its domains: the PH-like domain which has low affinity for PI(3)P, the J domain, and a YLT motif in the disordered C-terminus.
- This study defines a novel mechanism by which DNAJC13 function is regulated and defines the consequences of dysregulation of DNACJ13 activity on membrane protein recycling and endosomal distribution in cells.
