# Supplementary figures and images for: DNAJC13 localization to endosomes is opposed by its J domain and its disordered C-terminus
Source: Mol Biol Cell. Author manuscript; Available in PMC 2025 Sep 2. (PMC12404652; doi:10.1091/mbc.E24-12-0575)

Fig S1

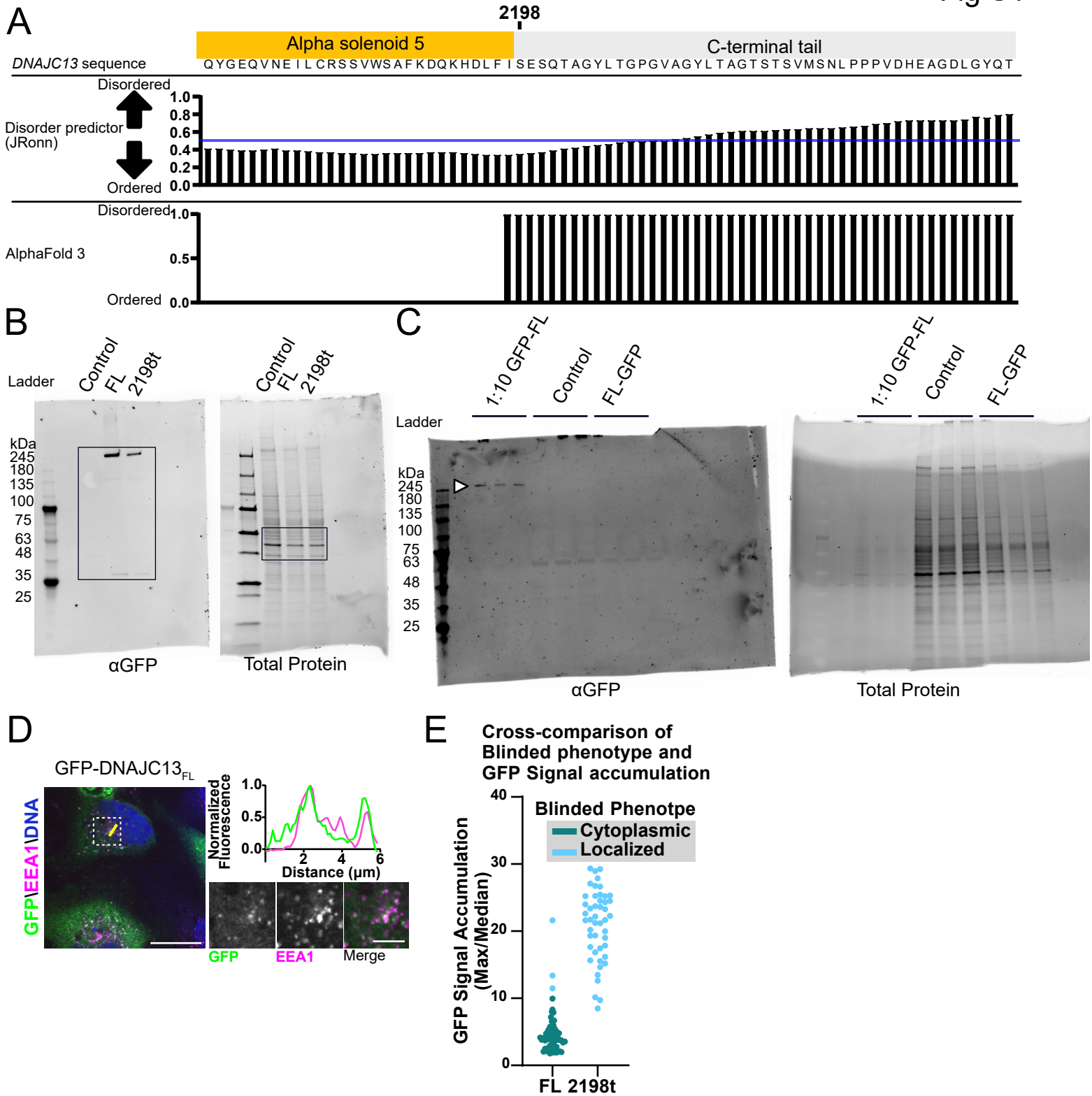

Supplement: Figure S1 — A, Structural prediction for the C-terminus of DNAJC13 (sequence, above), with JRonn disorder prediction (middle) and summary from five AlphaFold3 structural predictions (bottom). B, Uncropped anti-GFP western blot (left) and total protein stain gel (right) from Figure 1C; cropped area shown in the black box. C, Western blot (anti-GFP, left) and total protein stain (right) of extracts from three replicates of HeLa cells transfected with DNAJC13FL (at a 1:10 dilution of a standard load) or DNAJC13FL-ctGFP (undiluted), and a nontransfected control (Control). The white arrowhead points to DNAJC13. D, Fixed immunofluorescence microscopy image of GFP-DNAJC13FL expressed in HeLa cells. Imaged with anti-GFP (green), endosomal marker EEA1 (magenta), and DAPI DNA stain (blue) with insets shown to the right (scale bar = 20 μm, 5 μm in inset), (representative example from n=3 biological replicates). A line-scan (yellow line) showing normalized fluorescence intensity of GFP (green) and EEA1 (magenta) signal are plotted along the line (right). E, Cross-comparison of two phenotype assays, GFP signal accumulation metric data from Figure 1F, color coded by blinded phenotype analysis from Figure 1G. [file NIHMS2104826-supplement-Figure_S1.pdf]

Fig S3

A

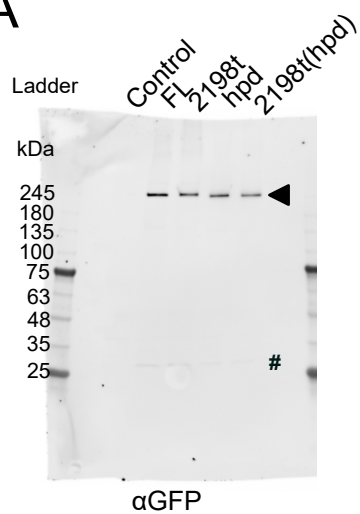

B

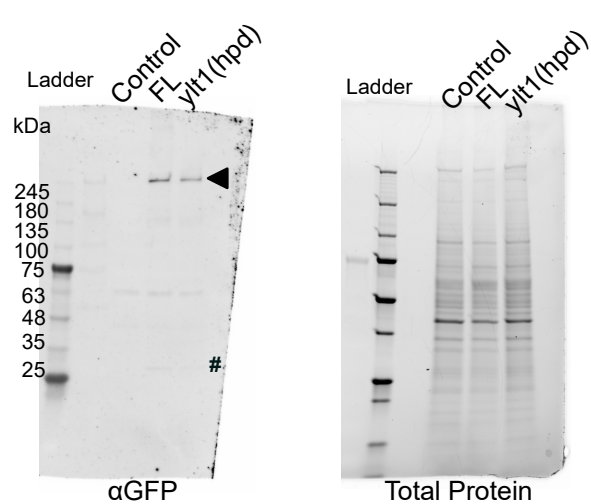

C

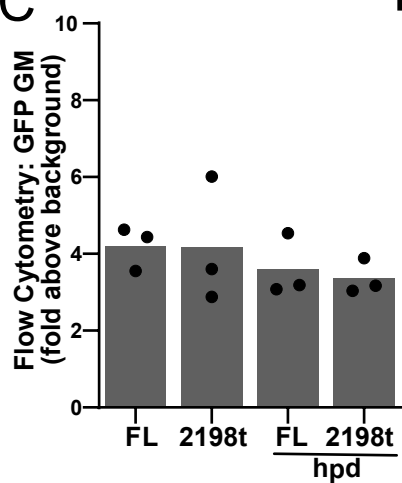

D

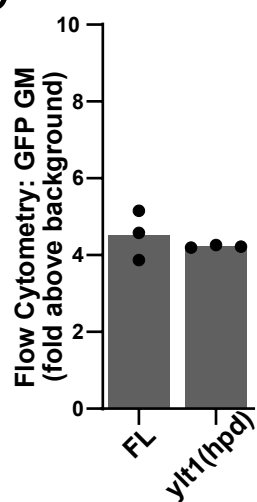

Supplement: Figure S3 — A, Representative western blot (anti-GFP, left) and total protein stain gel (right) of extracts from HeLa cells transfected with DNAJC13FL, DNAJC132198t, DNAJC13hpd, or DNAJC132198t(hpd) and a nontransfected control (Control) in HeLa cells (n=3 biological replicates). The arrowhead marks GFP-DNAJC13, and the # marks free GFP. B, Representative western blot (anti-GFP, left) and total protein stain gel (right) of HeLa cells transfected with DNAJC13FL, DNAJC13ylt1(hpd) and a nontransfected control (Control) in HeLa cells (n=3 biological replicates). The arrowhead marks GFP-DNAJC13, and the # marks free GFP. C, Flow cytometry-based expression analysis of DNAJC13FL, DNAJC132198t, DNAJC13hpd, and DNAJC132198t(hpd) in HeLa cells, assessed by geometric mean of GFP channel and displayed as fold above background signal from untransfected cells (n=3 biological replicates, bar represents mean). DNAJC13FL and DNAJC132198t data is the same as appears in 1B, all data acquired at same time. D, Flow cytometry-based expression analysis of DNAJC13FL or DNAJC13ylt1(hpd) in HeLa cells, assessed by geometric mean of GFP channel and displayed as fold above background signal from untransfected cells (n=3 biological replicates, bar represents mean). [file NIHMS2104826-supplement-Figure_S3.pdf]

Fig S2

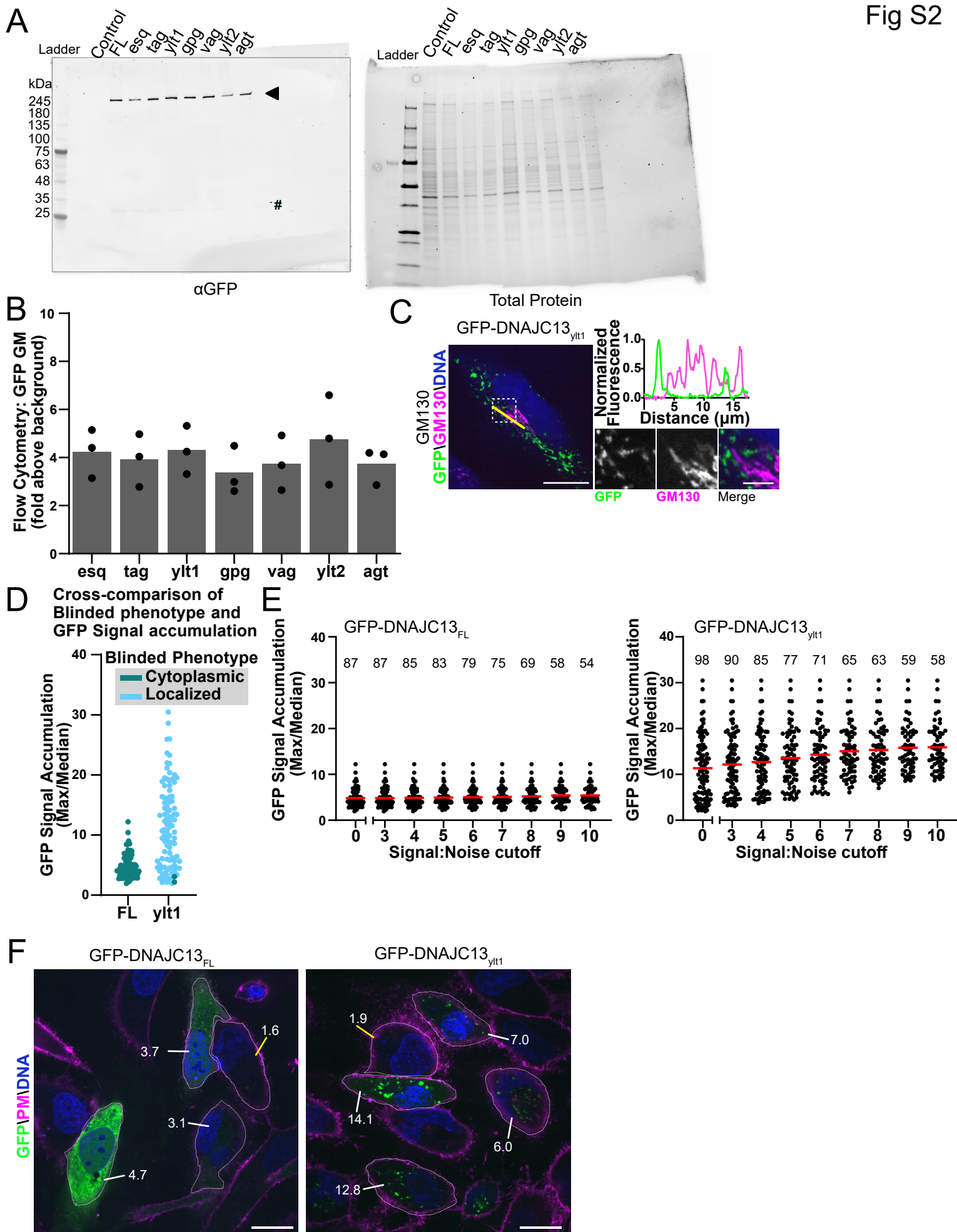

Supplement: Figure S2 — A, Representative western blot (anti-GFP, left) and total protein stain gel (right) of extracts from HeLa cells transfected with DNAJC13FL or triplet scanning mutants, and a nontransfected control (Control) (n=3 biological replicates). The arrowhead marks GFP-DNAJC13 and the # marks free GFP. B, Flow cytometry-based expression analysis of constructs expressed in HeLa cells, assessed by geometric mean of GFP channel and displayed as fold above background signal from untransfected cells (n=3 biological replicates, bar represents mean). C, Fixed immunofluorescence microscopy image of GFP-DNAJC13ylt1 expressed in HeLa cells. Imaged with anti-GFP (Green), Golgi marker anti-GM130 (magenta), and DAPI DNA stain (blue) with insets shown to the right (scale bar = 20 μm, 5 μm in inset), (representative example from n=3 biological replicates). A line-scan (yellow line) showing normalized fluorescence intensity of GFP (green) and GM130 (magenta) signal are plotted along the line (right). D, Cross-comparison of two phenotype assays, GFP signal accumulation metric data from Figure 2D, color coded by blinded phenotype analysis from Figure 2E. E, GFP signal accumulation metric data for DNAJC13FL and DNAJC13ylt1, previously shown in Figure 2D, with signal to noise cutoffs reveals lower population of DNAJC13ylt1-expressing cells by GFP signal accumulation scores are often low expressing cells. Numbers above datasets represent total number of cells in the dataset, red bars indicate the mean of the overall dataset. F, Representative images of DNAJC13FL and DNAJC13ylt1 containing high- and low-expressing cells in the same field of view. Cellular ROIs are drawn, with the GFP signal accumulation score annotated in white on the image (scale bar = 20 μm). In each image, a single non-expressing cell is analyzed, as annotated with the yellow line. [file NIHMS2104826-supplement-Figure_S2.pdf]

Fig S4

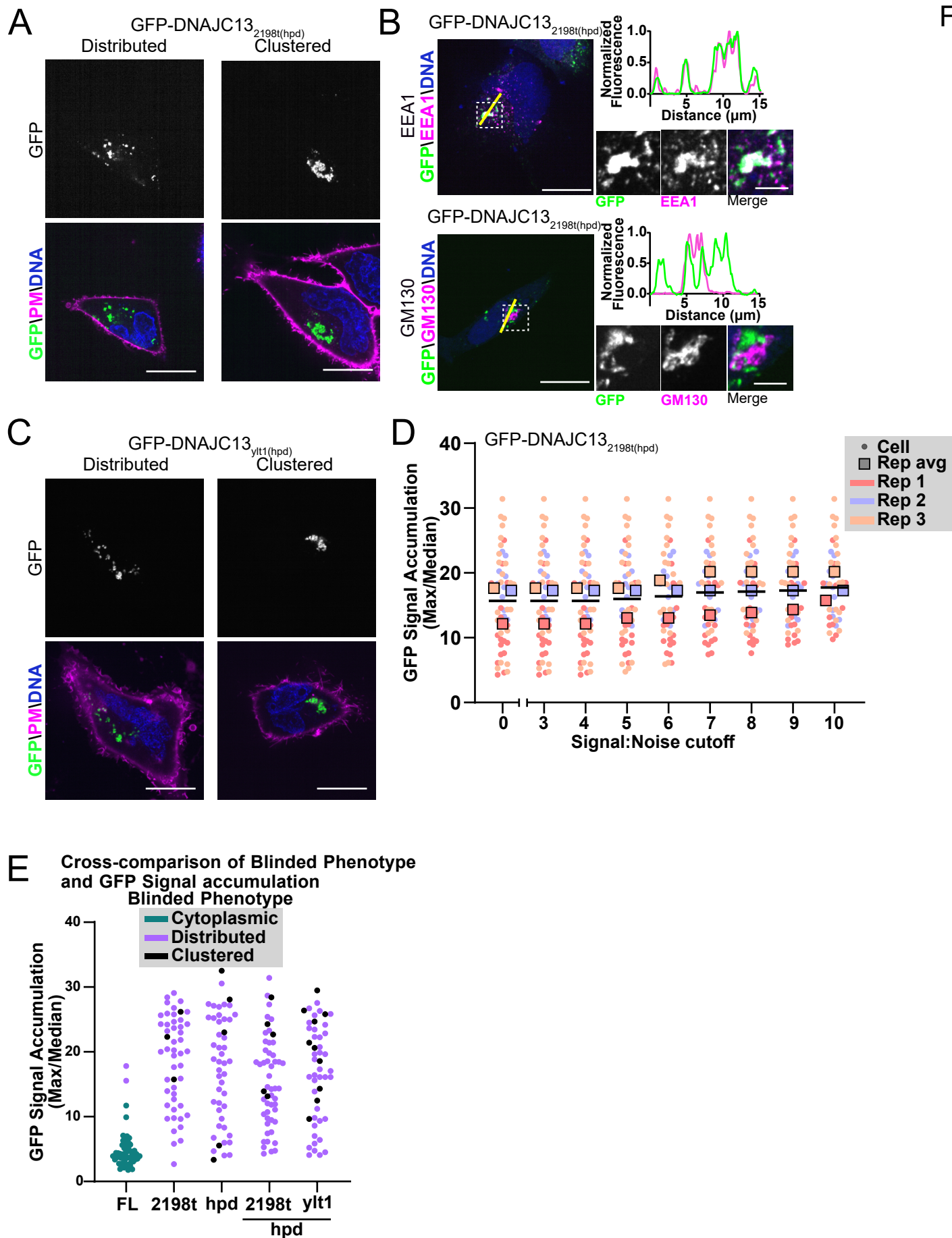

Supplement: Figure S4 — A, Live cell spinning disk confocal microscopy images of GFP-DNAJC132198t(hpd) in HeLa cells showing distributed (left) and clustered (right) endosomes. Imaged with CellMask plasma membrane stain (magenta) and Hoechst DNA stain (blue) (scale bar = 20 μm) (phenotypic representative examples from n=3 biological replicates). B, Fixed immunofluorescence microscopy image of GFP-DNAJC132198t(hpd) expressed in HeLa cells. Imaged with anti-GFP (Green), DAPI DNA stain (blue), and endosomal marker anti-EEA1 (magenta, top) or Golgi marker anti-GM130 (magenta, bottom). Insets shown to the right (scale bar = 20 μm, 5 μm in inset), (representative example from n=3 biological replicates). Line-scans (yellow lines) showing normalized fluorescence intensity of GFP (green) and EEA1 (magenta) or GM130 (magenta) signal are plotted along the lines (right). C, Live cell spinning disk confocal microscopy images of GFP-DNAJC13ylt1(hpd) in HeLa cells showing distributed (left) and clustered (right) endosomes. Imaged with CellMask plasma membrane stain (magenta) and Hoechst DNA stain (blue) (scale bar = 20 μm) (phenotypic representative examples from n=3 biological replicates). D, GFP signal accumulation metric data for DNAJC132198t(hpd), previously shown in Figure 3C, with signal to noise cutoffs for exclusion of data reveals one replicate with low signal to noise cells. Individual cells are shown as circles, with replicate averages shown in squares, data color coded by replicate, with black bars representing the average of the three biological replicate averages. E, Cross-comparison of two phenotype assays, GFP signal accumulation metric data from Figure 3C, color coded by blinded phenotype analysis from Figure 3D. [file NIHMS2104826-supplement-Figure_S4.pdf]

Fig S5

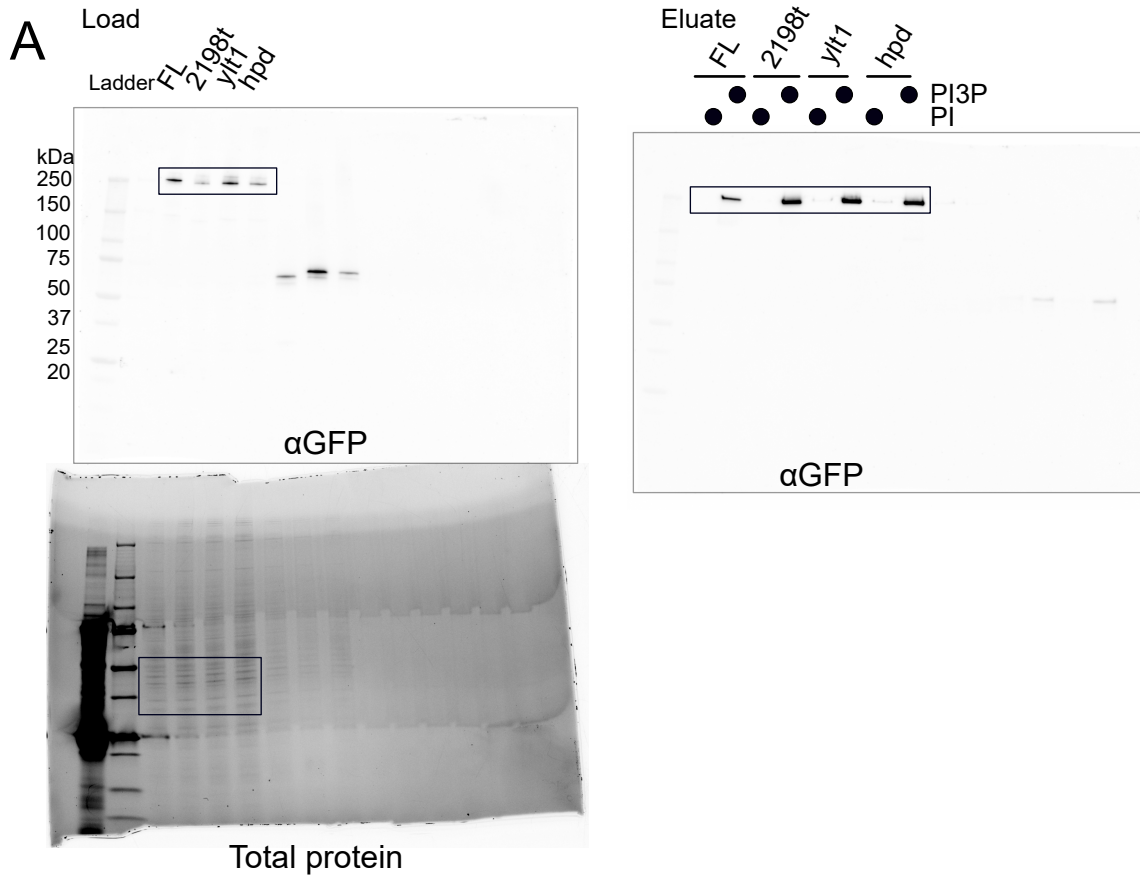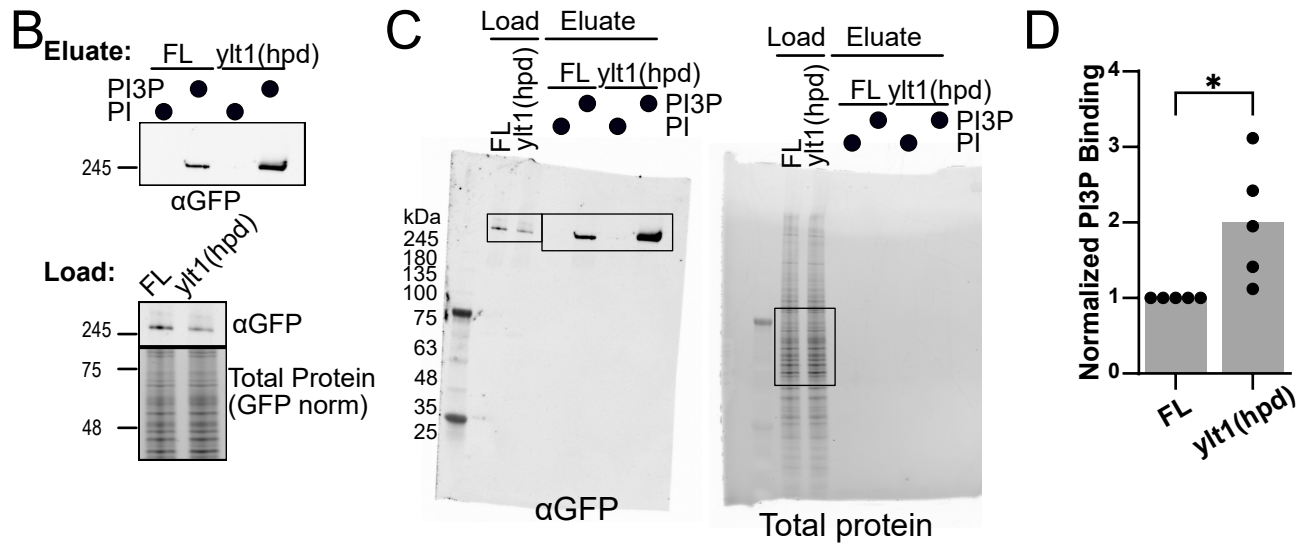

Supplement: Figure S5 — A, Uncropped blots (anti-GFP) and total protein stain gel from Figure 4A; cropped areas shown in the black boxes. Extra lanes are from a different experiment (see 5B/S7A). B, Western blots of PIP resin eluates for DNAJC13FL and double mutant GFP-DNAJC13ylt1(hpd). GFP-DNAJC13FL and GFP-DNAJC13ylt1(hpd) were expressed in HEK293 cells and lysates, normalized by flow cytometry for GFP expression, and were bound to PI (control) and PI(3)P decorated agarose resins. Loads and eluates were run on SDS-PAGE (load total protein stain, bottom) and immunoblotted for anti-GFP (load, middle; eluate, top). C, Uncropped blots (anti-GFP) and total protein stain gel from B. D, Quantification of PI(3)P pulldowns in B/C, normalized to load and the full-length pulldown (n=4 biological replicates, bar represents mean, paired two-tailed t-test, p=0.0481). [file NIHMS2104826-supplement-Figure_S5.pdf]

Fig S6

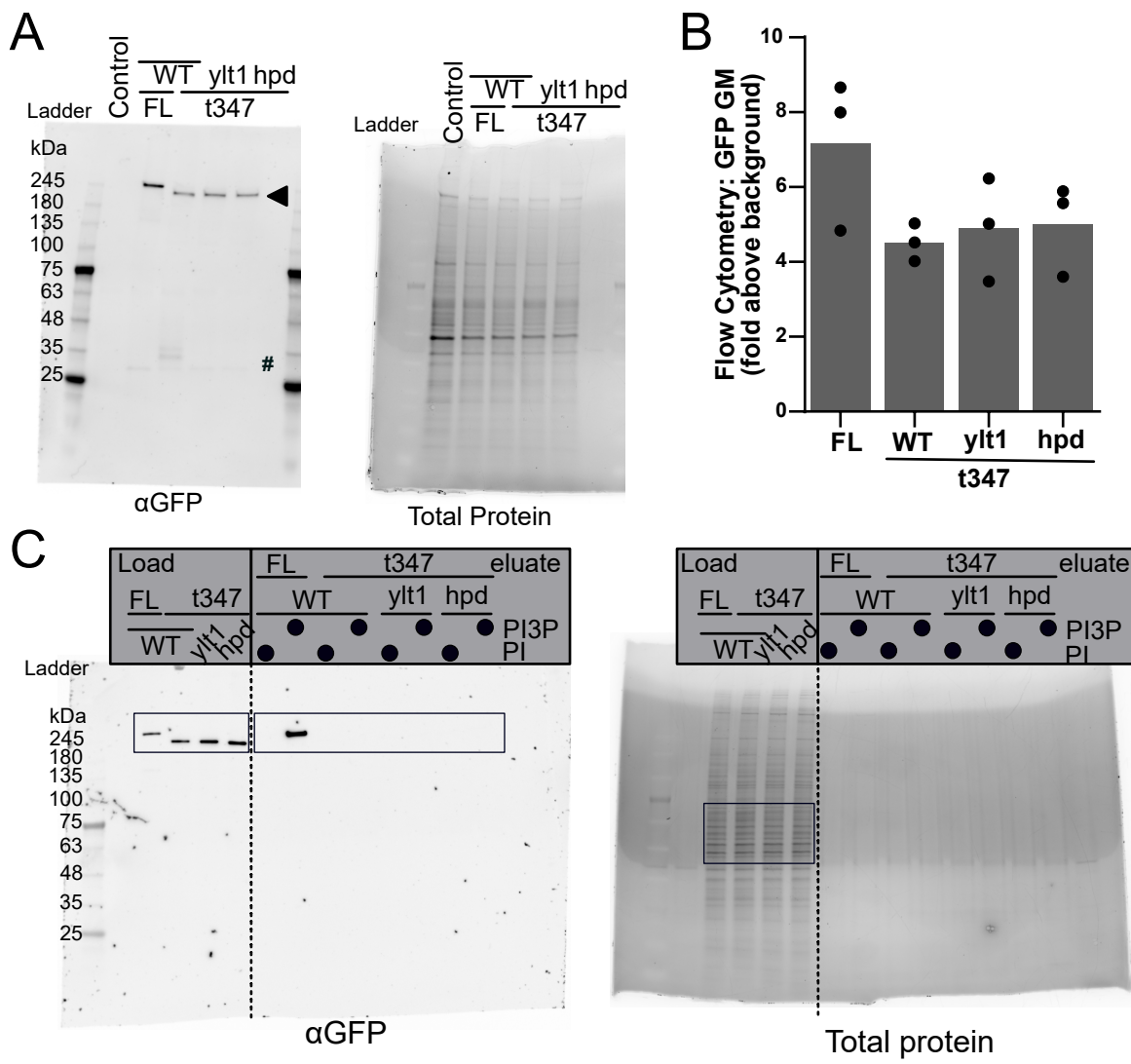

Supplement: Figure S6 — A, Representative western blot (anti-GFP, left) and total protein stain gel (right) of extracts from HeLa cells transfected with DNAJC13FL, DNAJC13t347, DNAJC13t347(ylt1), or DNAJC13t347(hpd), and a nontransfected control (Control) (n=3 biological replicates). Arrowhead marks GFP-DNAJC13 and the # marks free GFP. B, Flow cytometry-based expression analysis of t347 constructs in HeLa cells, assessed by geometric mean of GFP channel, displayed as fold above background signal from untransfected cells (n=3 biological replicates, bar represents mean). C, Uncropped blot (anti-GFP) and total protein stain gel from Figure 4C, cropped area shown in the black boxes. [file NIHMS2104826-supplement-Figure_S6.pdf]

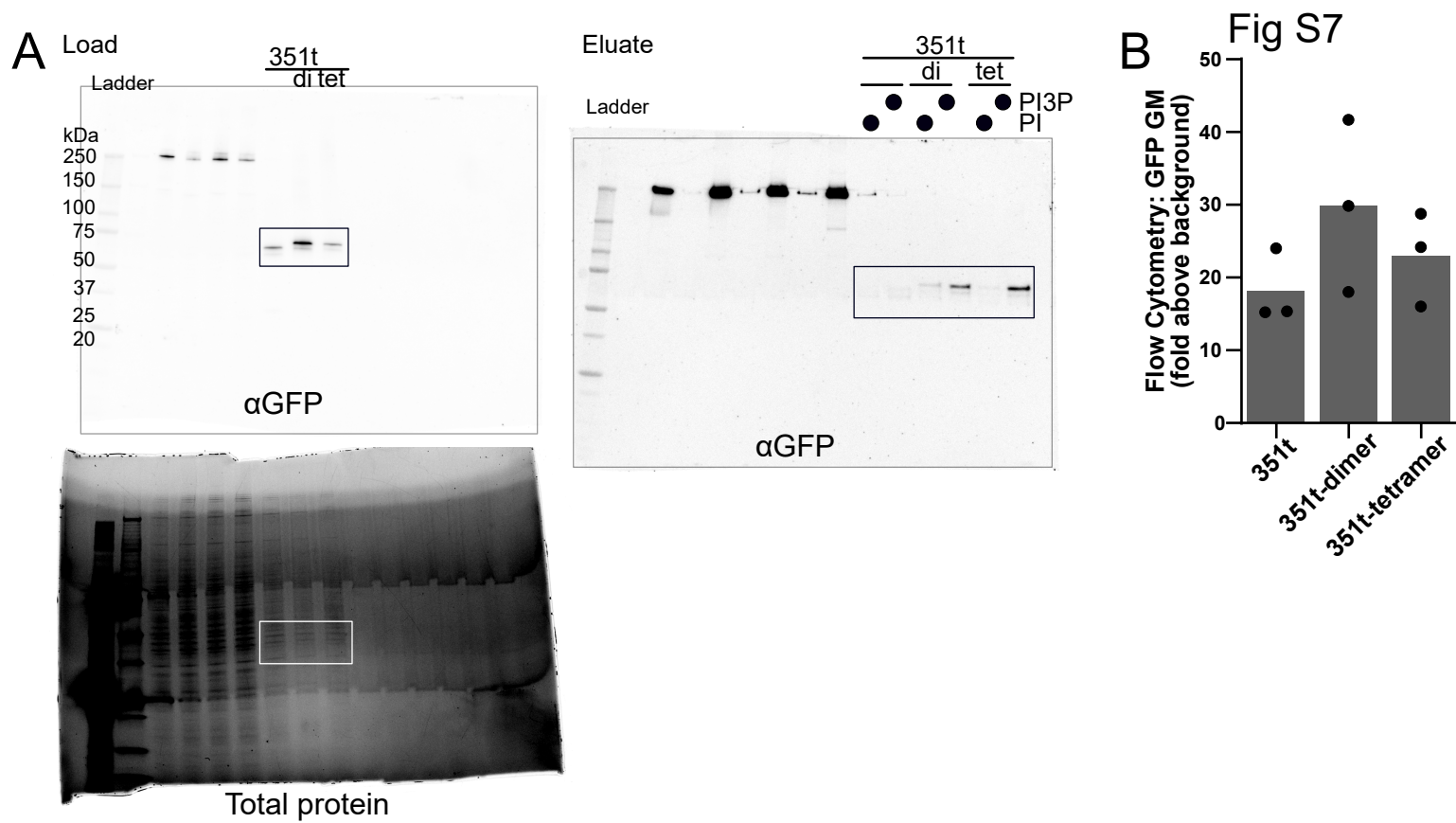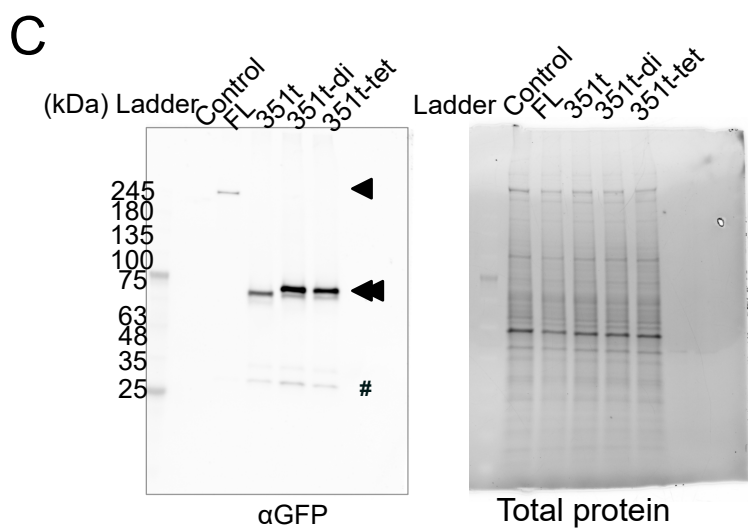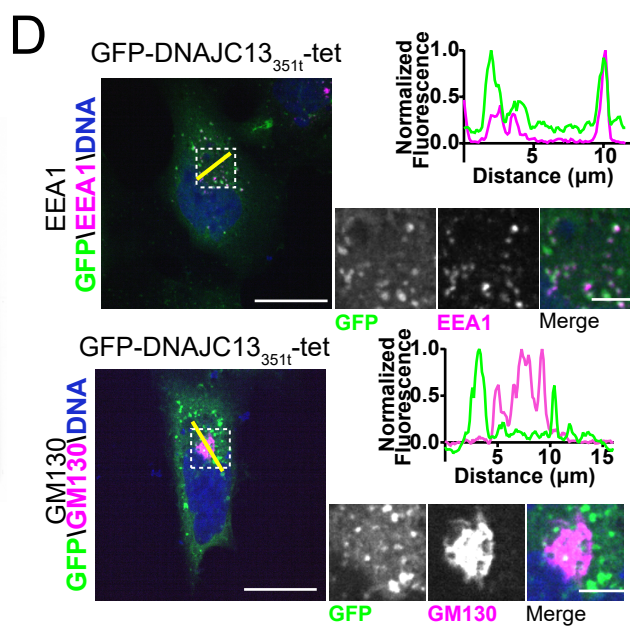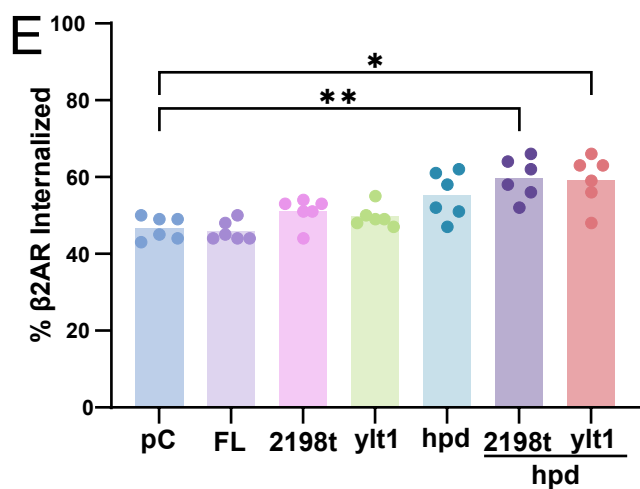

Supplement: Figure S7 — A, Uncropped blots (anti-GFP) and total protein stain gel from Figure 5B – gel was run with samples from Figure 4A, image was re-thresholded for viewing relevant samples, with cropped area shown in the black (or white) box. B, Flow cytometry-based expression analysis of DNAJC13351t constructs in HeLa cells, assessed by geometric mean of GFP channel, displayed as fold above background signal from untransfected cells (n=3 biological replicates, bar represents mean). C, Representative western blot (anti-GFP, left) and total protein stain gel (right) of extracts from HeLa cells transfected with DNAJC13351t, DNAJC13351t-dimer, or DNAJC13351t-tetramer, and a nontransfected control (Control) (n=3 biological replicates). Arrowhead marks GFP-DNAJC13FL, double arrowhead marks GPF-DNAJC13351t and the # marks free GFP. D, Fixed immunofluorescence microscopy image of GFP-DNAJC13351t-tetramer expressed in HeLa cells. Imaged with anti-GFP (Green), DAPI DNA stain (blue), and endosomal marker anti-EEA1 (magenta, left) or Golgi marker GM130 (magenta, right) with insets shown to the right (scale bar = 20 μm, 5 μm in inset), (representative example from n=3 biological replicates). Line-scans (yellow lines) showing normalized fluorescence intensity of GFP (green) and EEA1 (magenta) or GM130 (magenta) signal are plotted along the line (right). E, β2AR internalization induced by 30 minutes isoproterenol (10 μM) treatment measured 24 h after transfection with empty vector (pC) or DNAJC13 constructs. Cell surface receptor was measured by anti-FLAG-AF647 immunoreactivity and read out via flow cytometry in the APC channel (n=6 biological replicates, one-way paired ANOVA comparing all DNAJC13 constructs against empty vector with Dunnett’s multiple comparisons corrections, p= 0.0064 (DNAJC132198t(hpd)), 0.0229 (DNAJC13ylt1(hpd)), ns for all other comparisons. [file NIHMS2104826-supplement-Figure_S7.pdf]
